# Halogenated Cobalt Bis-Dicarbollide Strong Acids as Reusable Homogeneous Catalysts for Fatty Acid Esterification with Methanol or Ethanol

**DOI:** 10.3390/ijms252413263

**Published:** 2024-12-10

**Authors:** Pavel Kaule, Václav Šícha, Jan Macháček, Yelizaveta Naumkina, Jan Čejka

**Affiliations:** 1 Department of Chemistry, Faculty of Science, Jan Evangelista Purkyně University in Ústí nad Labem, Pasteurova 3632/15, 40096 Ústí nad Labem, Czech Republic; pavel.kaule@ujep.cz (P.K.); vaclav.sicha@ujep.cz (V.Š.); 2 Department of Syntheses, Institute of Inorganic Chemistry Czech Academy of Sciences, Hlavní 1001, 25068 Řež, Czech Republic; jmach@iic.cas.cz; 3 Department of Solid State Chemistry, Faculty of Chemical Technology, University of Chemistry and Technology, Technická 5, 16628 Prague, Czech Republic; yelizaveta.naumkina@vscht.cz

**Keywords:** cobalt bis-dicarbollide, sulphuric acid, acidity function, FAME, FAEE, esterification, green chemistry

## Abstract

The most commonly used homogeneous catalyst for fatty acid esterification is a corrosive sulphuric acid. However, this requires costly investment in non-corrosive equipment, presents a safety risk, is time consuming, and increases effluent generation. In this study, inorganic 3D heteroborane cluster strong acids are employed for the first time as homogeneous catalysts. Three novel isomeric tetrachlorido and tetrabromido derivatives of 3,3′-*commo*-bis[undecahydrido-*closo*-1,2-dicarba-3-cobaltadodecaborate](1−) [**1^−^**] were synthesised and fully characterised using a range of analytical techniques, including NMR, TLC, HPLC, MS, UV-Vis, melting point (MP), CHN analyses, and XRD. Ultimately, H_3_O[8,8′-Cl_2_-**1^−^**] was identified as the most efficient, reusable, and non-corrosive homogeneous catalyst for the esterification of four fatty acids. The reactions are conducted in an excess of alcohol at reflux. The effective absorption of water vapour provided by the molecular sieves maximises acid conversion. The hydrophobic dye Sudan black B was employed as an acid-base indicator to facilitate a comparison of the *H*_0_ acidity function of sulphuric acid and halogenated heteroboranoic acids when dissolved together in methanol. The ^23^Na NMR analysis demonstrated that the application of dry methanol resulted in the displacement of Na^+^ ions from zeolite, which subsequently exchanged the H_3_O^+^ ions of the acid. This process led to a gradual reduction in the efficiency of the catalysts, particularly with repeated use. The solution to this issue is to regenerate the catalyst on the ion exchanger following each reaction. In contrast to the published methods, our new approach meets 10 of 12 green chemistry principles.

## 1. Introduction

### 1.1. Sulfuric Acid-Catalyzed Esterification of Fatty Acids

Methyl and ethyl esters of fatty acids (FAMEs and FAEEs) are important liquid biofuels. The fundamental process of their production is the transesterification of fats and oils from various natural or waste sources, some of them contaminated with free fatty acids (FFAs). Inexpensive, locally available, inedible waste fats and oils suitable for second-generation biodiesel production often have elevated levels of FFAs [1,2,3,4]. While pure acylglycerols react efficiently with methanol to form FAMEs with the most common basic catalysts, namely KOH [5], NaOH [6], Me_4_NOH [7], or MeONa [8], free fatty acids readily form soaps with the basic catalysts. The quantitative conversion of low-FFA acylglycerols to FAMEs or FAEEs in less than 10 min under standard conditions was achieved after mixing with THF [5]. However, if the content of fatty acid salts exceeds approximately 1 wt.%, undesirable foam formation occurs, and the surface tension between the glycerine layer and the ester mixture is reduced. This, in turn, complicates the isolation of the product. Preprocessing of FFA-rich raw materials, as well as the transesterification itself, often leaves as a side product free or saponified fatty acids that can be further converted to FAMEs or FAEEs catalysed with strong mineral acids like HCl and H_2_SO_4_ [9].

Sulphuric acid has become the dominant homogeneous catalyst for the production of fatty acid methyl esters (FAMEs) from concentrated fatty acid mixtures obtained by saponification [3] or thermal decomposition of acylglycerols [10]. It has also been effectively used to pre-treat free fatty acids in vegetable oils using esterification before the transesterification of acylglycerols [4,11]. Since transesterification is also possible under acidic catalysis, albeit with a longer reaction time, biodiesel of the FAME type has also been prepared by the one-pot concurrent esterification of fatty acids and transesterification of triacylglycerols, diacylglycerols, and monoacylglycerols, both catalysed with sulphuric acid [12,13], in elevated yield and purity at the expense of greater energy consumption [12,14].

Esterification and hydrolysis of esters are complementary equilibrium reactions, as represented in the following scheme (1):

(1) R-COOH + R′-OH (H_3_O^+^) ⇌ R-COO-R′ + H_2_O fatty acid alcohol catalyst  biodiesel water

In order to shift the equilibrium in favour of the esters, it is necessary either to use a large excess of alcohol or to remove water from the reaction mixture. In industrial applications, water is often present in the raw materials used and should be removed beforehand. The mixture of fatty acid salts obtained by the saponification of acids and acylglycerols from soybean oil [11] was successfully preprocessed by freeze-drying with a falling film evaporator [15], while the water content of rubber seed oil could be lowered below 0.1% through simple heating for 10 min before the conditioned mixtures were treated with sulphuric acid and a ten-fold excess of methanol [16]. For FAEE synthesis, attempts to improve the esterification outcome with the use of anhydrous ethanol have also been made [17,18]. The addition of drying agents, like 175 wt.% of anhydrous MgSO_4_, directly to the reaction mixture with methanol and H_2_SO_4_ has also been tested [1]. It was observed that the most favourable outcomes under relatively mild reaction conditions were achieved by utilising a reactor equipped with a water absorber from boiling methanol [19] or ethanol [20,21,22] filled with activated 3Å zeolite molecular sieves.

Due to its favourable cost–benefit ratio, dehydrating properties, strength, and efficacy, H_2_SO_4_ has become the most widely used industrial homogeneous catalyst for the esterification of not only free fatty acids [23,24]. Using H_2_SO_4_ as a catalyst, successful esterifications have been reported even without any additional means of water removal from the reaction mixture, albeit with longer reaction times and lower yields of FAMEs (Table A1) [2,3,4,12,14,25,26,27,28] or FAEEs (Table A2) [10,29,30]. However, the dilution of H_2_SO_4_ in the course of the reaction results in a reduction in its catalytic effect [31,32,33], poses the risk of the corrosion of standard steel reactor equipment, complicates the recovery of the catalyst to the point it is not economically viable, and leads to the generation of significant quantities of harmful wastewater during the extraction or neutralisation process. Therefore, H_2_SO_4_, as a homogeneous catalyst for esterification, which is, in practice, consumed in a single use, demands increased investment costs for production equipment manufactured from corrosion-resistant materials and elevates the risk of occupational accidents. Furthermore, it is essential to monitor the residual sulphur content in the product to prevent corrosion in internal combustion engines [34].

There are many other fluid Brønsted–Lowry or Lewis acids (e.g., SO_2_·H_2_O, H_3_PO_4_, CH_3_SO_3_H) used to accelerate esterification reactions of free fatty acids [35]. Also, solid Brønsted–Lowry (e.g., Nafion NR50^®^, Amberlyst 15, 36, IR120H…), Lewis (e.g., AlCl_3_, FeCl_3_, TiOSO_4_, ZrOSO_4_, WO_3_-ZrO_2_, WO_3_-ZrO_2_-Al_2_O_3_, ionic liquids) acids or super-acids have been identified as heterogeneous esterification catalysts [31,36,37]. Insoluble in the reaction medium, the heterogeneous catalysts can be easily removed from the reaction mixture.

Unfortunately, prolonged and repeated stirring and manipulation with solid catalysts tend to wear the surface of the material and produce poorly isolatable powder residues [38]. Heterogeneous catalysts are usually micro- or mesoporous and have varying levels of strongly acidic or super-acidic groups on the surface. Unfortunately, in the course of the process, the large reactive surface gradually gets filled with the matrix. The ion exchange inhibits esterification by affecting the acidic group. The free acid centres are relatively easy to reactivate for reuse, but recovery from the pollution of the pores is usually much harder. Despite extensive progress in research, obstacles like these still hinder the replacement of established industrial applications of sulphuric acid with alternative technologies, and H_2_SO_4_ remains a dominant catalyst in laboratory and industrial esterification. The drawbacks of sulphuric acid use thus make the search for alternative catalysts still relevant.

The aim of this paper is to present, in particular, H_3_O[8,8′-Cl_2_-**1^−^**] as a new homogeneous, highly acidic but non-oxidizing, hydrophobic, very stable and reusable catalyst specifically used for the esterification of FFAs to FAMEs or FAEEs.

### 1.2. Methods for the Catalyst Acidity Determination

The strength of strong acids can be empirically categorised according to the Hammett acidity function *H*_0_, utilising a weak neutral base as a reference point:

(2) $$H_{0}=pK{\left({IH}^{+}\right)}_{aq}+log\frac{\left[I\right]}{\left[{IH}^{+}\right]}$$

In this context, p*K*(IH^+^)_aq_ represents the p*K_A_* value of the protonated form of the indicator in an aqueous solution. A variety of weakly basic aromatic compounds are employed as indicators, exhibiting distinct spectral absorption characteristics of the neutral indicator and its protonated form. The ratio of the concentrations of these two forms is determined using colorimetry [39]. The values of *H*_0_ are well uniform across a wide range of acid concentrations and are practically independent of the indicator used, whether neutral aromatic amines, azo compounds, ketones, or quinones. Furthermore, the *H*_0_ scale was demonstrated to be applicable to solutions in non-aqueous solvents or their mixtures with water. It should be noted that deviations from the values measured in aqueous solutions were observed, but rarely exceeded ±0.2. Electrical conductivity measurements and cryoscopy were employed to identify substances with a more negative *H*_0_ than 100% H_2_SO_4_, which were subsequently named super-acids. One such example is “magic acid” H[SbF_5_(SO_3_F)] [40].

The method of using a UV-Vis spectrometer to detect changes in the absorbance of a mixture of super-acids and a weak base dissolved in anhydrous HF was used [41]. Nevertheless, the strength of carborane acids, which are described as strong yet gentle super-acids, has yet to be determined on the *H*_0_ scale with the help of UV-Vis spectroscopy. This is due to the fact that they are solid rather than liquid phase acids, and thus cannot be accurately assessed using this method given their poor solubility in water. The acidity of the super-acids was evaluated and compared to that of other strong acids on the *Δδ* scale, which employs mesityl oxide as an indicator and for ^13^C NMR detection. This can be related to the *H*_0_ scale through the use of an empirical calibration curve [42,43,44]. The comparison of the strengths of solid and liquid phases represents a classic problem in physical chemistry. A universal thermodynamic acidity scale was proposed, based on the absolute chemical potential of the proton, using DFT calculations [45]. Despite the growing use of ionic liquids, the empirical *H*_0_ scale remains preferred, even when studying the solvent dependence of acidity in various acid-base systems [46].

In the field of green fuel development, the acidity of proposed catalysts represented by their *H*_0_ values was determined using various solvents, for example, DMSO [47] or ethanol [48]. It is essential to exercise caution when selecting both the solvent and the indicator when measuring the *H*_0_ of modern systems for acidic catalysis [49].

### 1.3. Heteroborane Strong Acids and Super-Acids

Previous research demonstrated that protonated halogenated derivatives of 12-vertex carborane, *closo*-CB_11_H_12_^−^, exhibit the strongest super-acid properties. These compounds are hygroscopic solids with extremely high thermal stability. Unlike sulphuric acid, fluorosulphuric acid, and other strong acids and super-acids, they are capable of protonating and forming salts of very weak bases, such as fullerene [50], C_60_, arenes [43], alkenes [51,52], SO_2_, CO, CO_2_, N_2_O, or NO_2,_ without any destructive oxidative effects [53].

**Figure 1:**
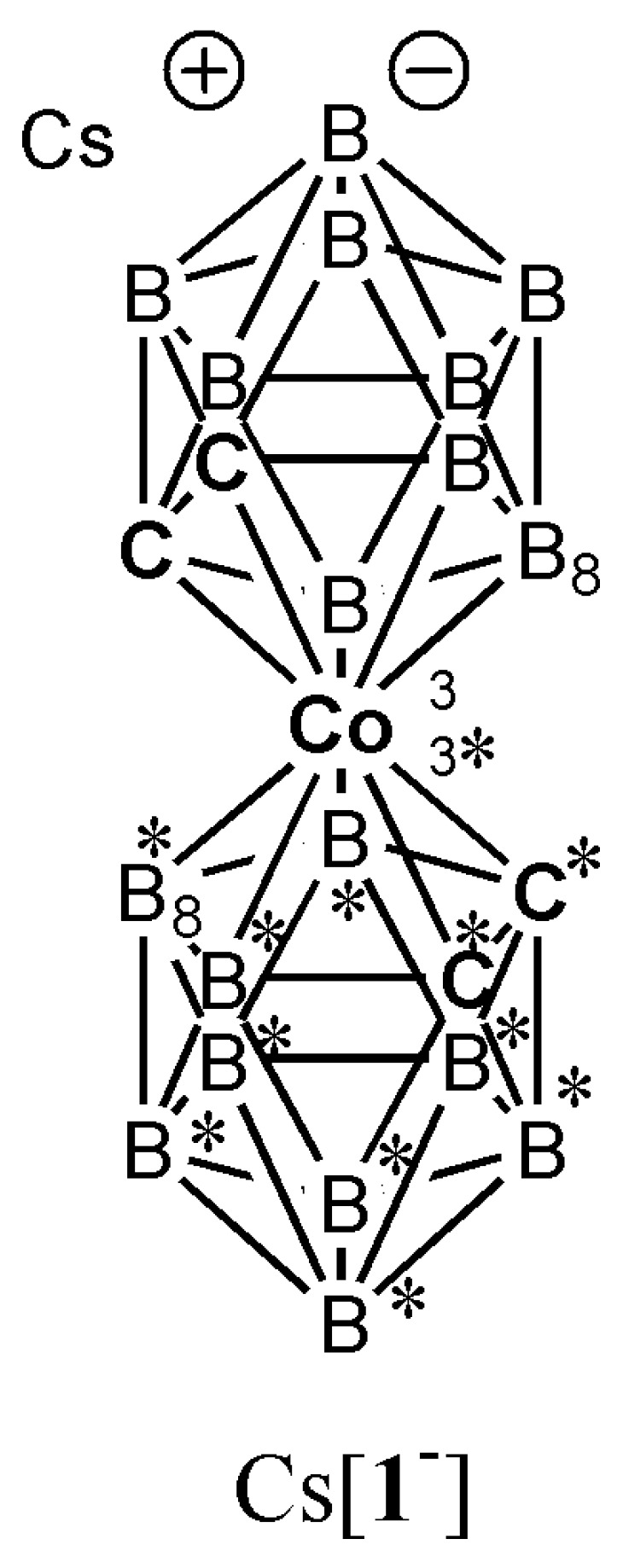
The structural formula of COSAN anion is 3,3′-*commo*-bis[undecahydrido-*closo*-1,2-dicarba-3-cobaltadodecaborate](1−) [**1^−^**]. The numbered vertices in the polyhedron represent the boron atoms. Each C or B atom is substituted terminally with one hydrogen atom. However, for clarity, cage terminal H atoms attached to numbered B and C vertices have been omitted [62]. The asterisk * has been used to clearly and visibly distinguish the cluster boron and carbon atoms in the lower cage. The positions of B8 (upper cage) and B8* (lower cage) are different.

Cesium salt of [**1^−^**], 18-electron π-complex 3,3′-*commo*-bis[undecahydrido-*closo*-1,2-dicarba-3-cobaltadodecaborate](1−), [(*closo*-1,2-C_2_B_9_H_11_)-3,3′*-commo-*Co^III^-(*closo*-1′,2′-C_2_B_9_H_11_)]^−^, is commonly referred to as cobalt bis-1,2-dicarbollide (Figure 1). This compound was first identified in 1965 [54] and is currently commercially available as a stable orange solid, marketed under the trade name COSAN.Cs. The compound structure belongs to the class of rigid heteroborane 3D clusters. Na[**1^−^**] exhibits amphiphilic and surface-active properties [55], low nucleophilicity, and weakly coordinated anion properties [56,57,58].

An orange solid H_3_O[**1^−^**] is distinguished by its extraordinary chemical, physical (UV), and radiolytical (α, β, γ) stability, as well as its solubility in mid-polar liquids (low mass alcohols, aldehydes, ketones, carboxylic acids, esters, amides, ethers, nitriles, nitro- and halogenated solvents). It should be noted that acid H_3_O[**1^−^**] is not soluble in water. However, the sodium salt of [**1^−^**] can be extracted from an organic, immiscible water phase [59]. Studies investigating the potential of H_3_O[**1^−^**] and its salts as extractants for the separation of nuclear fusion products have revealed that these compounds decompose relatively slowly, with half-lives of several days, under harsh conditions such as 3 M HNO_3_ at room temperature [60] or 1 M NaOH at elevated temperatures [61].

The initial halogen derivative [**1^−^**], designated [Br_6_-**1^−^**], has been described [63]. In the search for nuclear fission product extractants, halogen derivatives of [**1^−^**] were found to be highly stable, with over 60% of H_3_O[Br_6_-**1^−^**] and more than 80% of H_3_O[Cl_6_-**1^−^**] remaining intact after 36 days in 3M HNO_3_. The combination of such stability with the ability of [**1^−^**] and its derivatives to extract cations of alkali and alkaline earth metals from the aqueous to organic phase [59,64] has positioned halogen derivatives of [**1^−^**] as prospective reagents for the extraction of highly radioactive isotopes. ^137^Cs^+^ (B, t_1/2_ = 30.17 y) and ^90^Sr^2+^ (G, t_1/2_ = 29 y) can be extracted from high-level liquid waste following the previous extraction of Pu and U in the PUREX process [60,65]. In 1985, 300 kg of Cs[Cl_6_-**1^−^**] was prepared for this purpose at Spolana Neratovice n. p., Czechoslovakia, and the compound remains commercially available to this day [66].

A study published in 1982 led to the creation of a series of cobalt bis-dicarbollide anion derivatives that were uniquely physically and chemically stable. These derivatives were substituted on boron atoms with various numbers of chlorine, bromine, and iodine atoms. The radiation resistance of the parent [**1^−^**], dissolved in a mixture of methanol or nitrobenzene with CHBr_3_ or CCl_4_, was tested. The results demonstrated that halogenation of [**1^−^**] occurs, forming halogenated derivatives that exhibited enhanced radiative and oxidative stability relative to [**1^−^**]. The greater the applied dose in kGy, the greater the number of boron atoms in the cluster [**1^−^**] that have undergone stepwise substitution with halogen (8, 9, 12, 10…). The identical derivatives were then prepared using the standard laboratory procedure [67].

The synthesis of thermodynamically stable dihalogen derivatives [**1^−^**], in which the substituents are positioned symmetrically at positions 8 and 8′, with Cl, Br, and I, was achieved through a reaction with solid halogenating agents, specifically N-chloro- and N-bromosuccinimide [68], or via electrochemical synthesis [69]. These syntheses were conducted under standard conditions. The introduction of more than two halogen atoms, resulting in derivatives of lower symmetry, was achieved under more challenging conditions. However, this approach has consistently yielded mixtures of multiple products. The greater the number of equivalents of N-chlorosuccinimide employed, the greater the number of terminal hydrogen atoms on the boron atoms of the cluster [**1^−^**] that were substituted. The results of the experiments, in conjunction with the calculations of two-atom natural population analysis (2a-NPA), demonstrated that, as a consequence of kinetic influences, chlorination [**1^−^**] occurs with greater frequency at positions 8, 9, and 12 to Cl_6_ and at positions 7, 8, 9, and 12 to Cl_8_ derivatives [70].

To illustrate, the hexachloroderivative [**1^−^**] is the predominant species in a mixture containing trace amounts of penta- and heptachloroderivative. To date, only two polyhalogenated derivatives have been isolated in pure form at the analytical scale: [Cl_6_-**1^−^**] using isotachophoresis [71,72] and [Br_6_-**1^−^**] in the form of a single crystal [73]. A more complex three-step synthesis was employed to prepare several other boron-halogenated derivatives of [**1^−^**]. Initially, the parent *closo*-1,2-dicarbadodecaborane is halogenated. Subsequently, the products are deboronated under basic conditions to halogenated dicarbollide ligands. Finally, the sandwich complex with the Co^2+^ cation is formed [74,75,76]. In 2020, the first two C-halogenated derivatives of [**1^−^**], namely 1-Br and 1,1′-Br_2_ *rac*-isomer, were prepared by reaction with *n*-butyllithium (*n-*BuLi) and BrCN [77]. It was unexpected that C-substituted halogen derivatives of [**1^−^**] were not obtained during the analogous reaction with numerous other halogenation reagents. This is despite the fact that, following direct lithiation of [**1^−^**] with *n*-BuLi in previous studies, the use of -COOH, -OH, -ROH, RSH, -PR_3_, epoxides, cyclic ethers (up to 5 ring atoms), and S_8_ and PPh_3_ groups has resulted in the formation of the aforementioned compounds [78,79,80,81,82].

### 1.4. Metal Bis-Dicarbollide Complexes as Catalysts

Homogeneous Ziegler–Natta catalysts for the polymerisation and copolymerisation of ethylene and alpha-olefins in toluene or hexane have been synthesised from [Cp_2_ZrMe]^+^ and [PhNMe_2_H]^+^ and the weakly coordinating anion [(*closo*-1,2-C_2_B_9_H_11_)-3,3′-*commo*-M-(*closo*-1′, 2′-C_2_B_9_H_11_] (where M = Fe, Co, Ni) [83]. The lithium salt of [**1^−^**] was introduced as a highly efficient homogeneous catalyst for Mukaiyama–Michael reactions and for the substitution of allylic acetates by using a variety of nucleophiles [84,85]. The cesium salt of [(*closo*-1,2-C_2_B_9_H_11_)-3,3′-*commo*-Fe^+III^-(*closo*-1′,2′-C_2_B_9_H_11_)]^-^ was also employed as an effective heterogeneous catalyst for the combustion of pyroxilin propellant [86].

Salt of (4-CH_3_-(1-C_5_H_12_)-1-Py)^+^ [(*closo*-1,2,-(CH_3_)_2_-1,2-C_2_B_9_H_9_)-3,3′-Co^+III^-(1′,2′-(CH_3_)_2_-*closo*-1′,2′-C_2_B_9_H_9_)]^-^ was used as a cocatalyst and ionic liquid solvent for dissolution of lignin and oxidation of arylalcohols [87]. A mixed catalyst of PdAc_2_/[*n*-Bu_4_N][(1,2-(PPh_2_)-*closo*-1,2-C_2_B_9_H_9_)-3,3′-*commo*-Co^+III^-(1′,2′-(PPh_2_)-*closo*-1,2-C_2_B_9_H_9_)] allows 46% conversions (TON = 511) of diphenyl carbonate (DPC) to be obtained after 4 h at 110 °C during the oxidative carbonylation of phenol [88]. The potassium salt of a 2,3,9,10,16,17-hexakis(3-diethylaminophenoxy)-2,3-[(*closo*-1,2-C_2_B_9_H_11_)-3,3′-*commo*-Co^+III^-(*closo*-1′,2′-C_2_B_9_H_11_)]^−^ phthalocyaninato Zn^+II^ complex was used as an electrocatalyst for the reduction of hydrogen ions in an acidic medium in the HER process—enhanced hydrogen evolution reaction [89]. The reaction of ketones or aldehydes in a water solution with 0.01–0.1 mol% of Na[**1^−^**], Na[Cl_2_-**1^−^**], or Na[Cl_6_-**1^−^**] in the presence of UVA irradiation during the reaction provided yields of 90–99%, selectivity > 99%, and TON = 1000–10,000 [90].

Heterogeneous magnetically active Fe₃O_4_ nanoparticles with a covalently bonded catalyst, silylalkylammonium salt of [**1^−^**], were employed at a concentration of 0.1–0.01 mol% for UVA photooxidation of alkyl and aryl alcohols, resulting in yields of up to 99% and a TON of 1000 [91]. The water-soluble Na[**1^−^**] was introduced as a photoredox catalyst (0.1 mol%, TON = 1000) for the photooxidation of some alcohols in water and in 0.01 mol% (TON = 8500) for the oxidation of alkenes to epoxides. The employment of a complex cation and anion salt, namely [Ru^+II^(trpy)(bpy)(CH_3_CN)][**1^−^**]_2_, led to a further enhancement in the photooxidative conversion of alcohols to aldehydes or ketones at standard temperature. This was observed in an aqueous solution following UVA irradiation for up to 8 h (0.005 mol%, TON = 19–20,000) [92]. To the best of our knowledge, the cluster heteroboranoic acids, including [**1^−^**] and halogen derivatives of [**1^−^**], have not yet been investigated as Brønsted–Lowry acid catalysts for esterification. The objective of this study was to ascertain and contrast the strength of H_3_O[**1^−^**] acids and six monoanionic haloderivatives of [**1^−^**] with the protonation power of H_2_SO_4_. Additionally, their capacity to esterify a model mixture of four fatty acids (stearic, oleic, palmitic, myristic) with methanol and ethanol to FAMEs or FAEEs was evaluated.

## 2. Results and Discussion

### 2.1. General Method for Preparation of B-Halogenderivatives of Cs[**1^−^**]

The parent cluster Cs[**1^−^**] (250 mg, 0.55 mmol) was heated in a Schlenk flask with a magnetic stirrer in an oil bath to 200 °C in the vacuum of an oil pump for 2 h. Prior to use, 1,2-dimethoxyethane (DME, monoglyme) was subjected to a drying process with sodium, resulting in the formation of a dark blue sodium diphenyl ketyl complex. This complex was then distilled under dry conditions prior to the commencement of the reaction. Following the cooling of the reaction flask to room temperature and the introduction of argon as an inert atmosphere, the substance was fully dissolved in 25 cm^3^ of freshly distilled anhydrous DME. The flask containing the solution was cooled to −82 °C with the aid of a Julabo FT902 immersion cooler. Prior to use, the concentration of the active *n*-BuLi was verified in accordance with a published procedure [93]. Upon the addition of 1.10 mmol or 2.20 mmol of *n*-BuLi, the solution underwent a rapid change in colour from orange to dark purple. Subsequently, either two or four molar equivalents of the weighed solids F-TEDA and I_2_ or liquid bromine were added immediately using a syringe with a stainless steel needle. The chlorine gas was prepared by the reaction of hydrochloric acid with potassium permanganate, then dried using concentrated sulphuric acid and bubbled through the reaction mixture. Samples were periodically extracted from the reaction mixture and analysed with the help of mass spect33rometry (MS) and thin-layer chromatography (TLC) until a significant proportion of the desired derivative was achieved. Following the spontaneous heating of the reaction mixture in the room temperature (RT) flask, the volatiles and solvents were evaporated from the reaction mixtures on a rotary vacuum evaporator. The resulting residues were then dissolved in diethyl ether (50 cm^3^) and shaken twice with 3M HCl (50 cm^3^) and twice with distilled water (50 cm^3^). Subsequently, the diethyl ether solution was evaporated into a water emulsion. The emulsion was precipitated with a saturated CsCl solution, and the solid orange product was filtered on the glass frit and washed with three 50 cm^3^ portions of distilled water. The product was then dissolved in acetone (20 cm^3^), evaporated on a rotary evaporator, and dried in an oil bath (70 °C) under vacuum overnight.

### 2.2. Characterization of Prepared Derivatives of Cs[**1^−^**]

**Figure 2:**
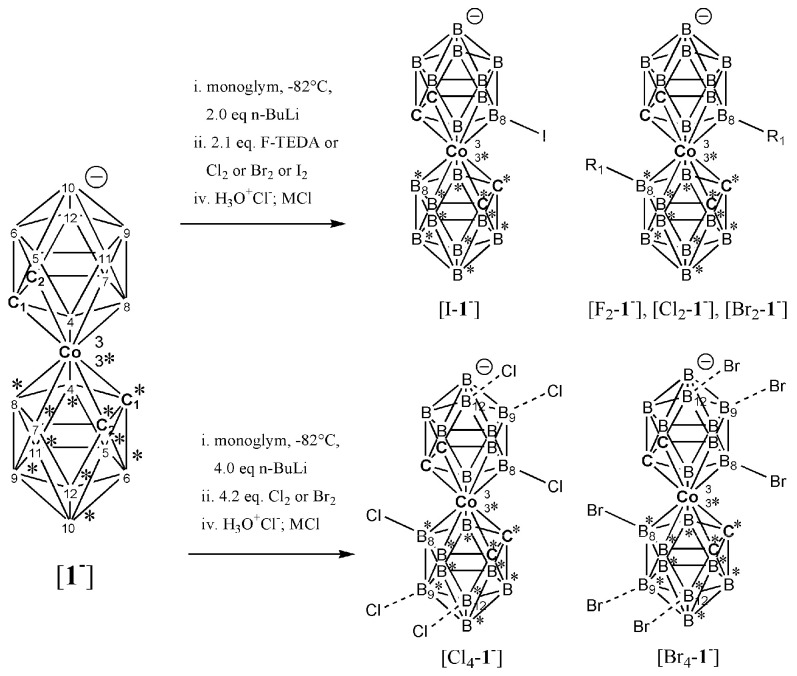
Reaction scheme of mono-, di-, tetrachlorido-, and tetrabromido derivatives of [**1^−^**] preparation. For clarity, cage terminal H atoms attached to numbered B and C vertices have been omitted. The asterisk * has been used to clearly and visibly distinguish the cluster boron and carbon atoms in the lower cage. The positions of B8 (upper cage) and B8* (lower cage) are different.

**Figure 3:**
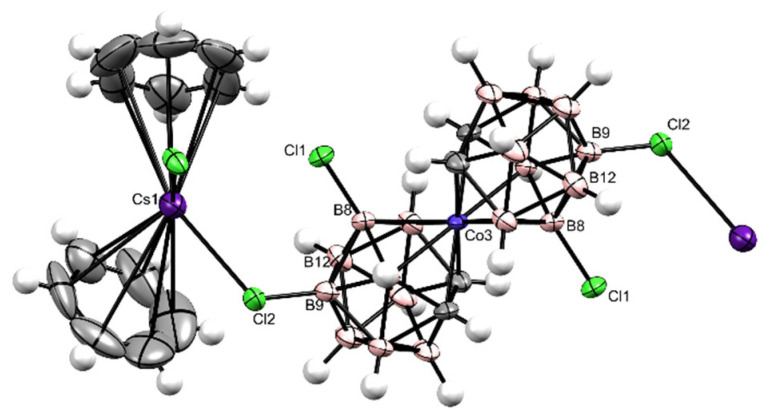
An ORTEP structure of the isomer Cs[8,8′, 9, 9′-Cl_4_-**1^−^**]·2 C_6_H_6_ determined using XRD of the single crystal.

**Figure 4:**
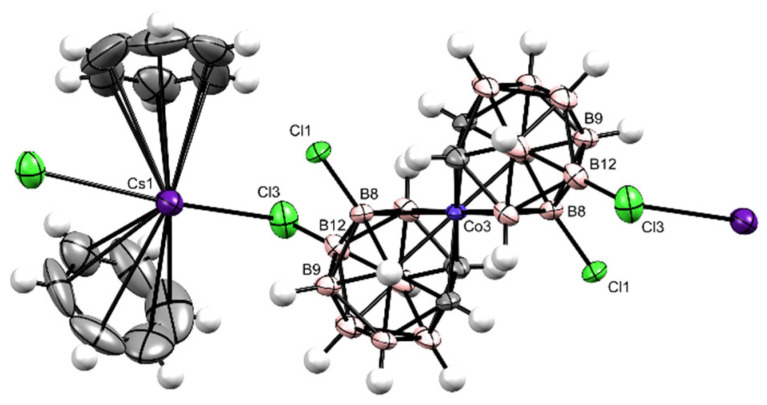
An ORTEP structure of the isomer Cs[8,8′,12,12′-Cl_4_-**1^−^**]·2 C_6_H_6_ determined using XRD of the single crystal.

The structural assignment of all prepared heteroborane salts, which were used in another step to prepare acidic catalysts, was mainly based on the knowledge of borane NMR spectra interpretation [94,95] and the use of HPLC-MS. ^1^H, ^11^B, ^13^C, ^19^F NMR and HPLC (Figure S10), ESI MS (Figure S11), and UV-Vis (Figure S12) confirmed that the substances K[F_2_-**1^−^**], Cs[Cl_2_-**1^−^**], Cs[8,8′,9,9′-Cl_4_-**1^−^**], K[Br_2_-**1^−^**], and Cs[I_1_-**1^−^**]·0.5 C_6_H_6_ are identical to previously prepared, isolated, and published compounds according to the previously described methods based on the application of H^+^/H_3_O^+^ Bronsted acid or X^+^ Lewis acid. In addition to the already known prepared halogen derivatives of [**1^−^**], three new, hitherto undescribed B-halogen derivatives [8,8′,12,12′-Cl_4_-**1^−^**] (Figure 2, Figure 3, Figure 4 and Figure S1), [8,8′,9,9′-Br_4_-**1^−^**], and [8,8′,12,12′-Br_4_-**1^−^**] (Figure 2 and Figure S1) were prepared by employing an unusual method for the preparation of B-substituted [**1^−^**] in an anhydrous strongly basic environment of *n-Bu*Li (Figure 1). The basic anhydrous reaction conditions apparently limit the degree and increase the regioselectivity of boron atom substitution alone. For example, a cluster of Cs[I_1_-**1^−^**] prepared by the reaction of Cs[**1^−^**] with iodine in methanol [67] at RT leads to a formation of 16% of Cs[I_2_-**1^−^**] as a side product. After the reaction in anhydrous monoglym with *n-Bu*Li, only trace amounts of Cs[I_2_-**1^−^**] were identified with the help of LC-MS (Figure S10). The defined high purity of all hydrophobic salts prepared was confirmed by CHN elemental combustion analysis (Table S1).

Successfully prepared single crystals of Cs[Cl_4_-**1^−^**] allowed the structure of very similar positional isomers 47% [8,8′,9, 9′-Cl_4_-**1^−^**] (Figure 3) and 47% [8,8′,12, 12′-Cl_4_-**1^−^**] (Figure 4) of Cs[Cl_4_-**1^−^**] to resolve accompanied by a small quantity of 6% less symmetric derivatives of Cs[Cl_5_-**1^−^**]—[7,8,8′,9,9′-Cl_5_-**1^−^**] and [7,8,8′,12,12′-Cl_5_-**1^−^**] (Figure S20—^1^H signals at 4.469 and 4.197 ppm; Figure S21—^13^C signals at 54.932 and 47.411 ppm) using XRD (Figure 3 and Figure 4), HPLC (Figure S4), MS (Figure S5), UV-Vis (Figure S6), and NMR (Figures S20–S24) methods. Also, a solid Cs[Br_4_-**1^−^**] was recognized as a proportional 1:1 mixture of [8,8′,9,9′-Br_4_-**1^−^**] and [8,8′,12,12′-Br_4_-**1^−^**] coupled with no more than 6% of pentabromide Cs[Br_5_-**1^−^**] isomers [7,8,8′,9,9′-Br_5_-**1^−^**] and [7,8,8′,9,9′-Br_5_-**1^−^**] using HPLC (Figure S7), MS (Figure S8), UV-Vis (Figure S9), and NMR (Figures S25–S29) methods. Both these positional isomer mixtures containing mostly Cs[Cl_4_-**1^−^**] and Cs[Br_4_-**1^−^**] are very difficult to isolate, but the samples were well defined for use as acidic catalysts. Solid hygroscopic heteroborane acids H_3_O[F_2_-**1^−^**], H_3_O[Cl_2_-**1^−^**], H_3_O[Cl_4_-**1^−^**], H_3_O[Br_2_-**1^−^**], H_3_O[Br_4_-**1^−^**], and H_3_O[I_1_-**1^−^**] · 0.5 C_6_H_6_ were prepared from the respective hydrophobic salts after evaporation of ethanol from the solution passed over the H_3_O^+^ activated Amberlyst15.

The hydrophobicity of halogen derivatives of [**1^−^**] was measured on a nonpolar C8 reverse phase column using HPLC. The results indicated a gradual increase in hydrophobicity in the series from K[F_2_-**1^−^**] > Cs[I_1_-**1^−^**] · 0.5 C_6_H_6_ > Cs[Cl_4_-**1^−^**] > Cs[Cl_2_-**1^−^**], with the most hydrophobic Cs[Br_4_-**1^−^**]. It was determined that the entirety of the halogen derivatives enumerated in Table S2 are highly stable, permanent anions (70–114 eV) following the application of a fragmentation energy established during the CID experiment. This outcome aligns with prior findings regarding the elevated chemical and physical stability of these heteroboranes [60].

Cs[8,8′,12,12′-Cl_4_-**1^−^**] (Figure 3, Figure 4, and Figure S1) Cesium 8,8′,12,12′-tetrachlorido-3,3′-*commo*-bis[undecahydrido-*closo*-1,2-dicarba-3-cobaltadodecaborate](1−) present in the 1:1 mixture with known Cesium 8,8′,9,9′-tetrachlorido-3,3′-*commo*-bis[undecahydrido-*closo*-1,2-dicarba-3-cobaltadodecaborate](1−) Cs[8,8′,12,12′-**1^−^**] were both accompanied by no more than 6% of assymetrical impurities 8′,9′-dichlorido-[undecahydrido-*closo*-1′,2′-dicarba-3′-cobaltadodecaborate]-3′-*commo*-3-7,8,9-trichlorido-[undecahydrido-*closo*-1,2-dicarba-3-cobaltadodecaborate](1−) [7, 8,8′, 9,9′,Cl_5_-**1^−^**], 8′,12′-dichlorido-[undecahydrido-*closo*-1′,2′-dicarba-3′-cobaltadodecaborate]-3′-*commo*-3-7,8,12-trichlorido-[undecahydrido-*closo*-1,2-dicarba-3-cobaltadodecaborate](1−) [7, 8,8′, 12,12′,Cl_5_-**1^−^**].

Results: Yield 61.5% (0.34 mmol); MS ESI *m*/*z* for B_18_Co_1_C_4_ Cl_4_H_18_: 461.16 (100%) [M − H]^−^, calcd.: 461.12 (100%); TLC: Rf = 0.4; HPLC: Rt = 3.08 min., UV λmax = 319 nm; MP: <300 °C; NMR: δ ^11^B (192 MHz, CD_3_CN, Et_2_O.BF_3_) 10.95 s (2B, *B*8,8′-Cl_2_), 2.90 s (2B, *B*12,12′*-*Cl_2_), −0.18 d (2B, *J =* 138, *B*10,10′), −5.85 m (4B, *J =* 147, *B*4′,7′,4,7,), −7.86 d (2B, *J =* 136, *B*9,9′-Cl_2_), −18.81 d (2B, *J =* 161, *B*5′,11′), −20.44 d (2B, *J =* 164, *B*5,11), −25.84 d (2B, *J =* 174, *B*6,6′); δ ^1^H (600 MHz, CD_3_CN, Me_4_Si) 4.40 s (1H, C*Hcarb*), 4.36 s (1H, C*Hcarb*), 4.33 s (1H, C*Hcarb*), 4.29 s (1H, C*Hcarb*), 3.09 s (4H, B(4,7,4′,7′)*H*), 3.01 s (2H, B(10,10′)*H*), 3.01 s (2H, B(9,9′)*H*), 2.09 s (2H, B(5′,11′)*H*), 1.68 s (1H, B(6)*H*), 1.68 s (2H, B(5,11)*H*), 1.68 s (1H, B(6′)*H*);δ ^13^C (150 MHz, CD_3_CN, Me_4_Si) 52.17 d(2C, *J =* 180, *C*carb), 50.32 d(2C, *J =* 178, *C*carb). Crystallographic data. *M* = 377.4 g·mol^−1^, monoclinic system, space group *C* 2/*c*, *a* = 26.1684(7) Å, *b* = 8.7069(2) Å, *c* = 16.7097(4) Å, *α* = 90°, *β* = 122.8341(8)°, *γ* = 90°, *Z* = 4, *V* = 3199.00(14) Å^3^, *D_c_* = 1.567 g·cm^−3^, *μ*(Cu-Kα) = 16.169 mm^−1^, crystal dimensions of 0.100 × 0.207 × 0.304 mm.

Cs[8,8′,9,9′-Br_4_-**1^−^**] (Figure S1) Cesium 8,8′,9,9′-tetrabromido-3,3′-*commo*-bis[undecahydrido-*closo*-1,2-dicarba-3-cobaltadodecaborate](1−) present in the 1:1 mixture with Cs[8,8′,12,12′-Br_4_-**1^−^**] (Figure S1) Cesium 8,8′,12,12′-tetrachlorido-3,3′-*commo*-bis[undecahydrido-*closo*-1,2-dicarba-3-cobaltadodecaborate](1−), accompanied by assymetrical impurities 8′,9′-dibromido-[undecahydrido-*closo*-1′,2′-dicarba-3′-cobaltadodecaborate]-3′-*commo*-3-7,8,9-tribromido-[undecahydrido-*closo*-1,2-dicarba-3-cobaltadodecaborate](1−) [7, 8,8′, 9,9′,Br_5_-**1^−^**], 8′,12′-dibromido-[undecahydrido-*closo*-1′,2′-dicarba-3′-cobaltadodecaborate]-3′-*commo*-3-7,8,12-tribromido-[undecahydrido-*closo*-1,2-dicarba-3-cobaltadodecaborate](1−) [7, 8,8′, 12,12′,Br_5_-**1^−^**].

Results: Yield 42.55% (0.23 mmol); MS ESI *m*/*z* for B_18_ Br_4_Co_1_C_4_H_18_: 639.92 (100%) [M − H]^−^, calcd: 639.92 (100%); Rf = 0.36; HPLC: Rt = 4.15 min., UV λ_max_ = 326 nm; MP: <300 °C, NMR: δ ^11^B (192 MHz, CD_3_CN, Et_2_O.BF_3_) 5.23 s (2B, *B*8,8′-Br_2_), 1.21 d (2B, *J =* 150, *B*10,10′), −3.01 s (2B, *B*9, 9′ or *B*12, 12′-Br_2_), −3.59 s (1B, *J =* 138, *B*4), −4.97 m (3B, *J =* 152, *B*7,9,9′ or *B*7,12,12′-Cl_3_), −6.58 m (2B, *J =* 157, *B*4′,7′), −17.97 d (2B, *J =* 185, *B*5′,11′), -19.13 d (2B, *J =* 201, *B*5,11), −24.65 d (2B, *J =* 173, *B*6,6′); δ ^1^H (600 MHz, CD_3_CN, Me_4_Si) 4.56 s (1H, C*Hcarb*), 4.54 s (1H, C*Hcarb*), 4.49 s (1H, C*Hcarb*), 4.47 s (1H, C*Hcarb*), 3.33 s (1H, B(4)*H*), 3.43 s (1H, B(7)*H*), 3.22 s (2H, B(10,10′)*H*), 3.10 s (2H, B(4′,7′)*H*), 2.28 s (4H, B(5′,11′,9,9′)*H*), 1.87 s (2H, B(6,6′)*H*), 1.78 s (2H, B(5,11)*H*); δ ^13^C (150 MHz, CD_3_CN, Me_4_Si) 55.59 d (2C, *J =* 180, *CH*carb), 53.57 d(2C, *J =* 182, *CH*carb).

### 2.3. Acidity Function Determination

**Figure 5:**
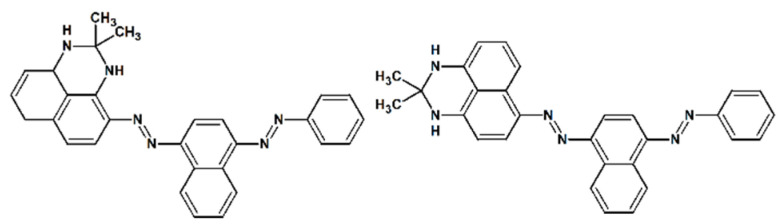
The structural formulas of commercial Sudan Black B dye components.

**Figure 6:**
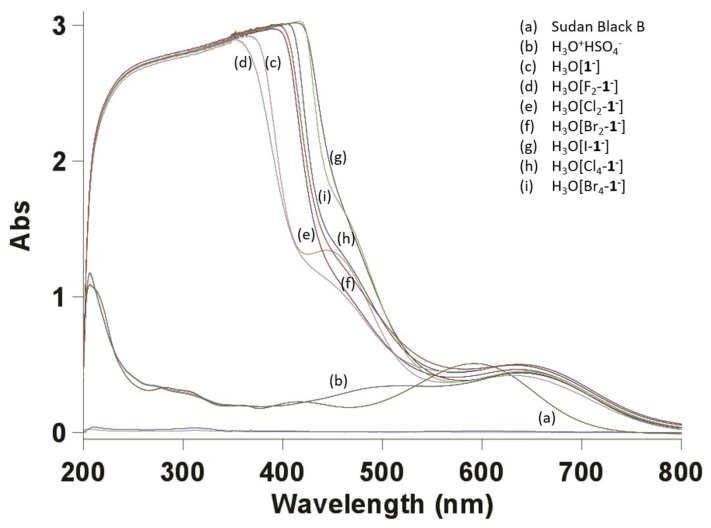
The overlapped UV-Vis spectra of all used acids (5 × 10^−3^ mol) after reaction with the Sudan Black B (III) indicator (7.2 × 10^−5^ mol) were measured in pure methanol at 25 °C. The other collected UV-Vis spectra have been included in the Supplementary Information (Figures S13 and S14).

The diazodiimino polycyclic dye Sudan Black B (III), which contains two protonizable nitrogen atoms (Figure 5), was selected as a weak aromatic base for the indicator (7.2 × 10^−5^ mol). Sudan Black B is a mixture of two principal components, designated as Sudan Black B I and Sudan Black B II. Sudan Black B I is 2,2-dimethyl-1,3-dihydroperimidin-4-yl)-(4-phenylazo-1-naphthyl)diazene, while Sudan Black B II is 2,2-dimethyl-1,3-dihydroperimidin-6-yl)-(4-phenylazo-1-naphthyl)diazene [96]. The Sudan Black B (III) highlights the instability of the dye, particularly at pH values below 1.1 [97]. All of the heteroborane catalysts studied, including Sudan Black B, are coloured hydrophobic substances that are soluble in methanol or a mixture of 75% methanol and 25% water. The absorption bands of the indicator and catalysts do not overlap, allowing for the reliable measurement of changes in the indicator’s absorbance due to its protonation with strong acid (Figure 6).

The acidity of the heteroborane acids under examination is evidently similar to that of H_2_SO_4_ but not significantly higher (Table 1). Consequently, these heteroborane compounds cannot be unambiguously designated as super-acids. It can be observed that in the case of H_3_O[Cl_2_-**1^−^**] dissolved in 75% methanol, the calculated *H*_0_ value is, in fact, more negative than the *H*_0_ value of 98 wt.% sulphuric acid dissolved in the same molar concentration. The studied homogeneous catalysts exhibit the characteristics of strong, non-oxidising yellow-orange hydrophobic acids. It is noteworthy that the acidity of the cluster [**1^−^**] remains relatively unaffected by halogen substitution.

### 2.4. Esterification of Four Fatty Acids Mixture in Methanol or Ethanol

**Figure 7:**
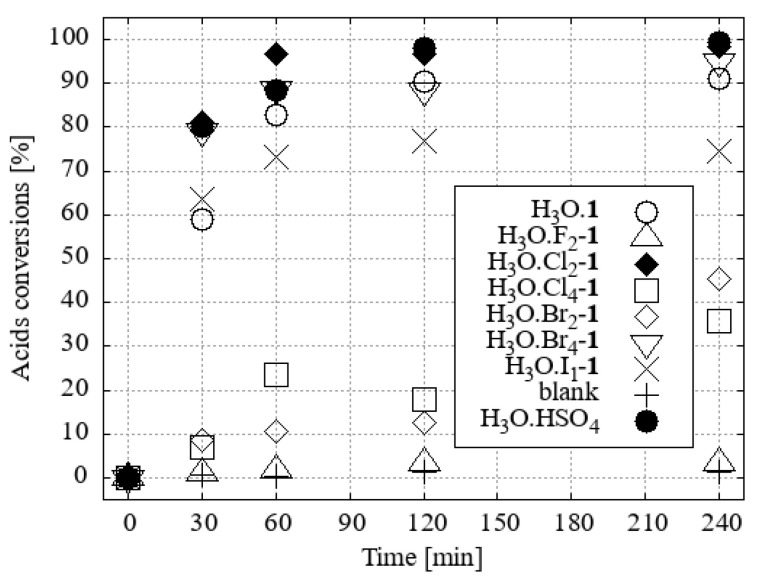
The conversion of fatty acid methyl esters (FAMEs) to their corresponding acid using methanol as the solvent and 1 mol% of the catalyst under desiccated conditions was investigated.

**Figure 8:**
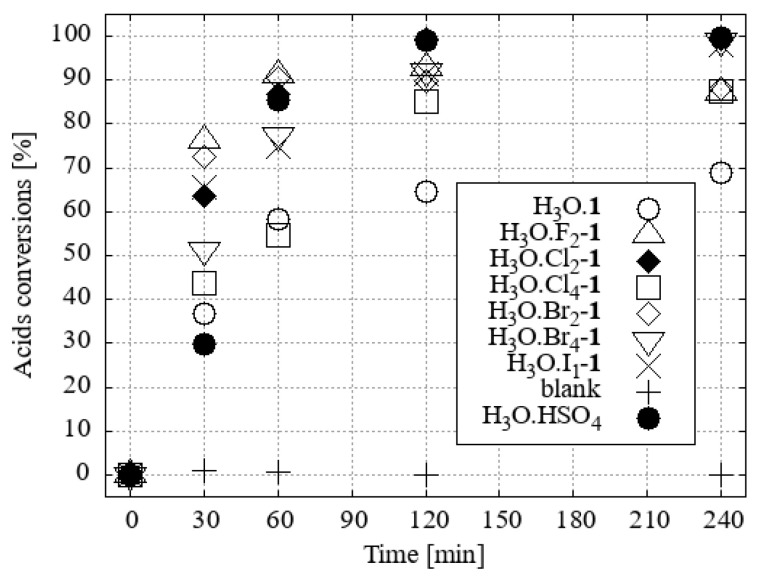
The conversion of acid to FAEEs with ethanol and a 1 mol% catalyst under desiccation conditions was investigated.

Esterification reactions were carried out under identical conditions (N_2_ atmosphere, reaction temperature, time, vapour cooling, agitation) in a parallel synthesis system, Carousel 6Plus (Radleys & Co., Ltd., Saffron Walde, UK), in flasks (50 cm^3^) with a vertical glass tube surrounded by a central aluminium, cold water-cooled cooler. A vertical tube was filled with 10 g of desiccant, namely activated 3Å zeolite molecular sieves, which primarily served as an absorber of water vapour from the boiling mixture. The published experimental results indicate that the removal of water from the vapour phase of methanol [19] or ethanol [20] azeotropes is a slower process than the direct addition of molecular sieves or other desiccants to the reaction mixture. However, this method allows for seamless alternating regeneration of the desiccant. A solution of a mixture of four fatty acids (0.86 mmol of each, namely myristic, palmitic, oleic, and stearic acids) was prepared in a reaction flask by dissolving the mixture in a solvent-reactant (5 cm^3^; 123.4 mmol of desiccated methanol or 85.6 mmol of ethanol). The initial H_3_O[**1^−^**] and its hydroxonium homologue derivatives [F_2_-**1^−^**], [Cl_2_-**1^−^**[Br_2_-**1^−^**], [I_1_-**1^−^**], [Cl_4_-**1^−^**], and [Br_4_-**1^−^**] were subjected to testing in a molar ratio of 3 (0.15 mmol; Figures S16 and S17), 2 (0.10 mmol; Figures S18 and S19), or 1 mol % (0.05 mmol; Figure 7 and Figure 8) with respect to the fatty acid content. The same concentrations of H_2_SO_4_ under the same conditions were used for comparison of the esterification activity.

Liquid microsamples (40 mm^3^) were taken from each boiling mixture at 0, 30, 60, 120, and 240 min. Following the addition of 1.5 mm^3^ of a CH_3_CN/CHCl_3_ mixture (1:1, *v*/*v*), the samples were analysed using HPLC APCI MS. The two-segment MS method (Figure S15) enables direct comparison of the sample composition via the TIC (total ion current) method, utilising the difference between the area integrals of separated fatty acids and their esters (FAMEs or FAEEs). Following the distillation of alcohol, the highly pure product, FAMEs or FAEEs, was consistently separated from the reaction mixture using vacuum distillation in a vacuum of 0.001 mbar at an oil bath temperature of 200–210 °C. The distillation residue contains a non-volatile anionic heteroborane with residues of unreacted fatty acids. Analyses of the obtained FAME/FAEE products (HPLC, UV-Vis, MS) revealed that traces of catalysts were never present. Furthermore, expected oxidation products of fatty acids, particularly oleic acid, were not detected in any of the HPLC MS chromatograms.

The preparation of FAMEs with 3 mol. % of heteroboranes was most efficiently catalyzed with H_3_O[Br_2_-**1^−^**] within 30 min. The second most effective catalyst was H_3_O[Cl_2_-**1^−^**] (see Figure S16). The order of reactivity has remained relatively consistent in the case of reactions with 2 mol% of catalysts. The conversion rate exceeded 95% within 30 min when H_3_O[Br_4_-**1^−^**] and H_3_O[**1^−^**] were employed as catalysts (Figure S18). 

It was found that the efficiency of the H_3_O[Br_4_-**1^−^**] catalyst in methanol decreased slightly when only 1 mol% was used. H_3_O[Cl_2_-**1^−^**] became the most efficient catalyst in this series (Figure 7).

For the reaction in ethanol, H_3_O[F_2_-**1^−^**] and H_3_O[Cl_2_-**1^−^**] proved to be the two most effective catalysts (Figure 8). 

**Figure 9:**
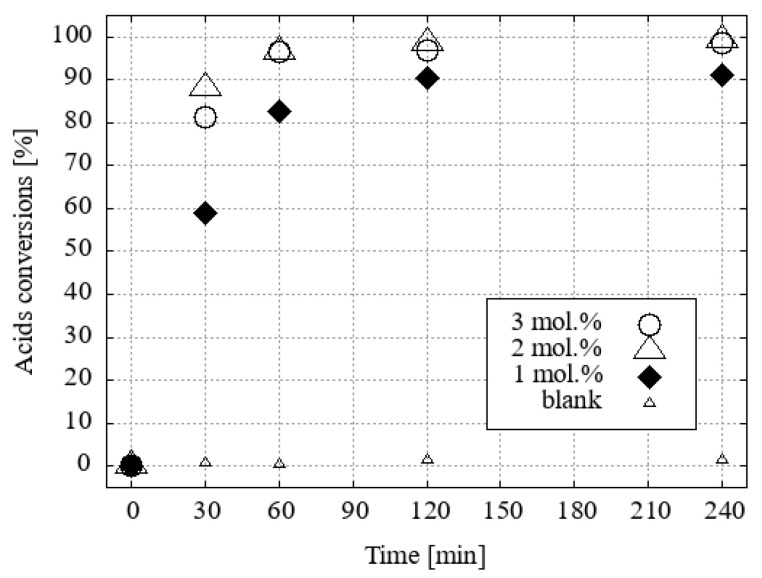
Acids conversions of FAMEs with methanol and various mol. % of the H_3_O[Cl_2_-**1^−^**] under desiccation.

Therefore, after an extensive series of experiments, H_3_O[Cl_2_-**1^−^**] was identified as the most readily available and durable catalyst to complete the optimisation of the required amount of catalyst for esterifications carried out in methanol and ethanol. Surprisingly, the use of 1 mol% H_3_O[Cl_2_-**1^−^**] in methanol resulted in a maximum conversion of 90% (Figure 9). 

**Figure 10:**
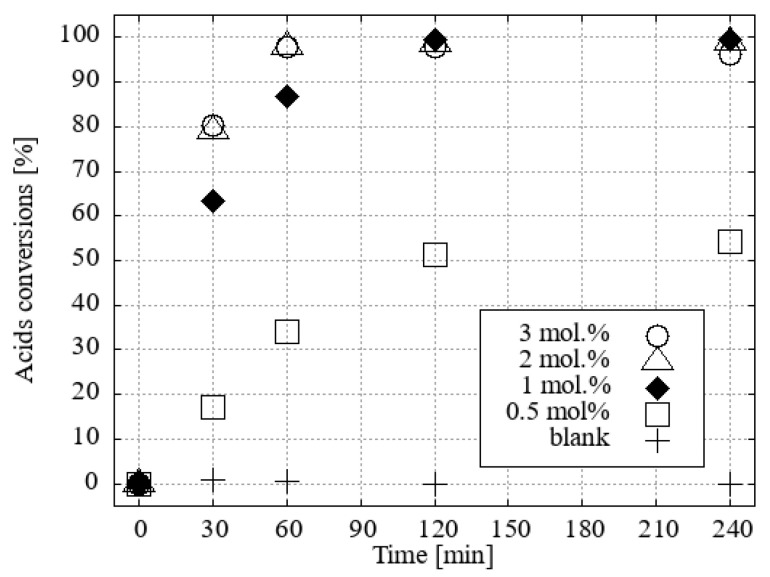
Acids conversions of FAEEs with ethanol and various mol. % of the H_3_O[Cl_2_-**1^−^**] under desiccation.

Since a similar reaction in ethanol resulted in more than 95% conversion of acids to FAME, the use of only 0.5 mol% catalyst was also tested. Here the limiting concentration for the use of H_3_O[Cl_2_-**1^−^**] was apparently reached (Figure 10).

**Figure 11:**
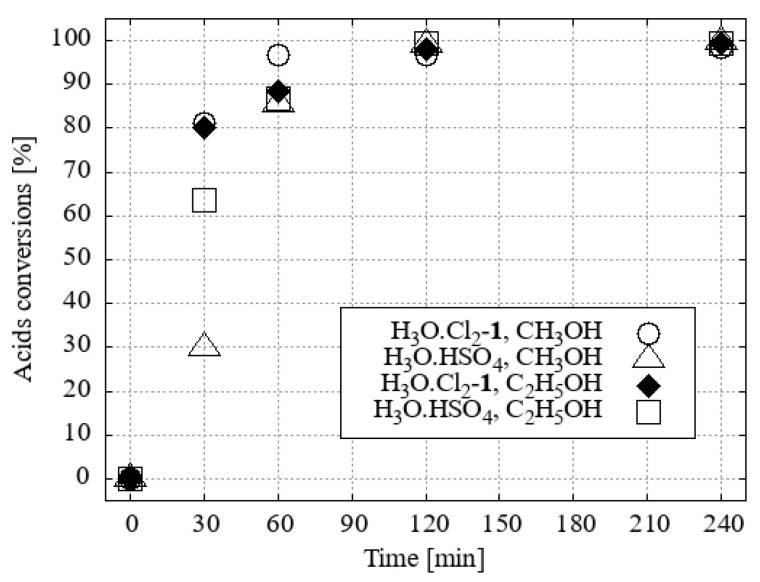
Acids conversions of FAMEs and FAEEs with 1 mol. % of the catalyst under desiccation.

Comparison of the efficiency of 1 mol. % of H_3_O[Cl_2_-**1^−^**] and H_3_O.HSO_4_ in the esterification of fatty acids in methanol and ethanol showed that H_3_O[Cl_2_-**1^−^**] was more efficient in both cases (Figure 11).

Table A1 presents an overview of the key parameters for FAMEs, while Table A2 provides a similar overview for FAEEs. These tables allow for a comparison of the number of reactants, catalysts, time, and acid conversions between strong acids, H_3_O[Cl_2_-**1^−^**] catalyst and H_2_SO_4_. The review demonstrates that the use of heteroborane catalysts in conjunction with an excess of alcohol and the continuous absorption of water vapours formed in the process allows for the production of both FAMEs and FAEEs under mild reaction conditions within a single hour, with a high yield. The removal of water from the reaction mixture appears to be a crucial factor in increasing the esterification conversions of free fatty acids.

**Figure 12:**
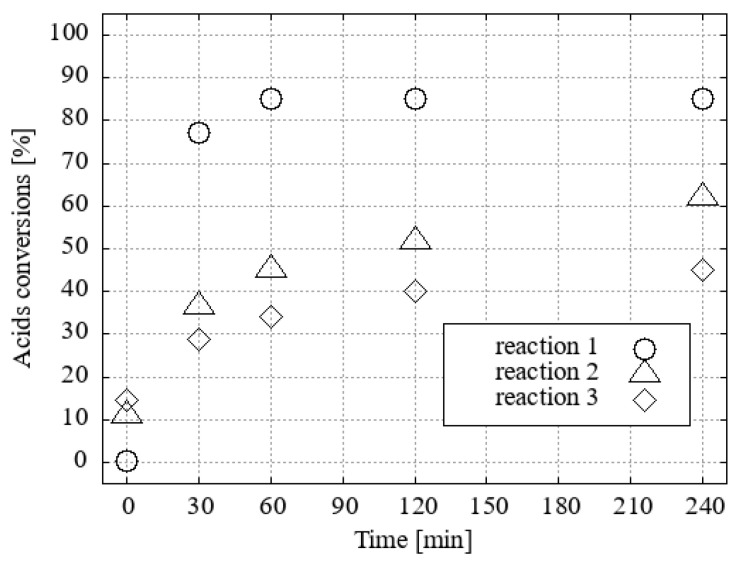
Repeatability of fatty acid conversions into FAMEs after 3 reactions with 1 mol.% of H_3_O[Cl_2_-**1^−^**] without reactivation treatment.

**Figure 13:**
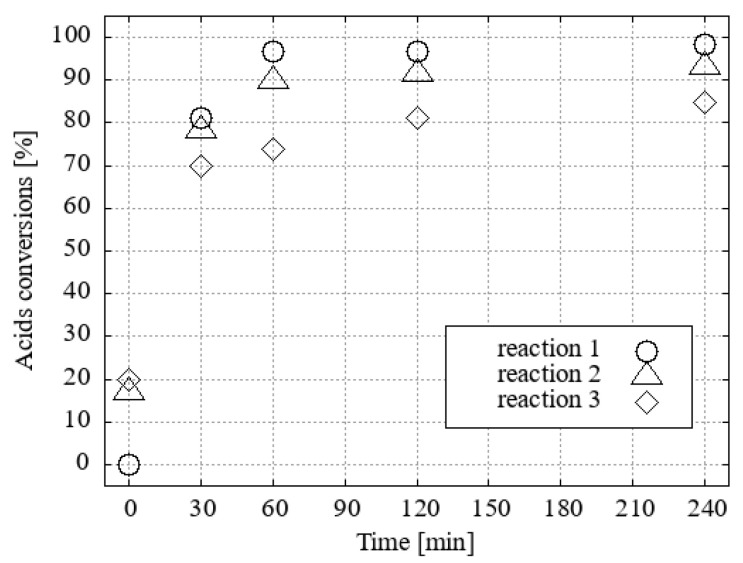
Repeatability of fatty acid conversions into FAEEs after 3 reactions with 1 mol.% of H_3_O[Cl_2_-**1^−^**] without reactivation treatment.

A noticeable trend of inhibition was observed in the recovery of 1 mol.% H_3_O[Cl_2_-**1^−^**] and the repeatability of the catalytic process. This is supported by data from the repeated catalytic tests conducted after methanol (Figure 12) or ethanol (Figure 13) evaporation and distillation of esters.

The difference in acid conversions with methanol between the first and third reactions without proton activation of the catalyst was 40%, in comparison to 15% with proton activation. With regard to acid conversions with ethanol, the discrepancy was 10% in the absence of H^+^ activation of the catalyst, whereas only 1% was observed with the latter. This progressive loss of catalytic activity, which was more pronounced in methanol than in ethanol, was attributed to the partial neutralisation of the acidic catalyst. The release of sodium ions from our 3Å molecular sieves—zeolites—was demonstrated through the use of ^23^Na NMR (see Figures S30–S33), which revealed that the vapours of wet alcohol, particularly methanol, gradually release sodium ions (Table 2).

**Figure 14:**
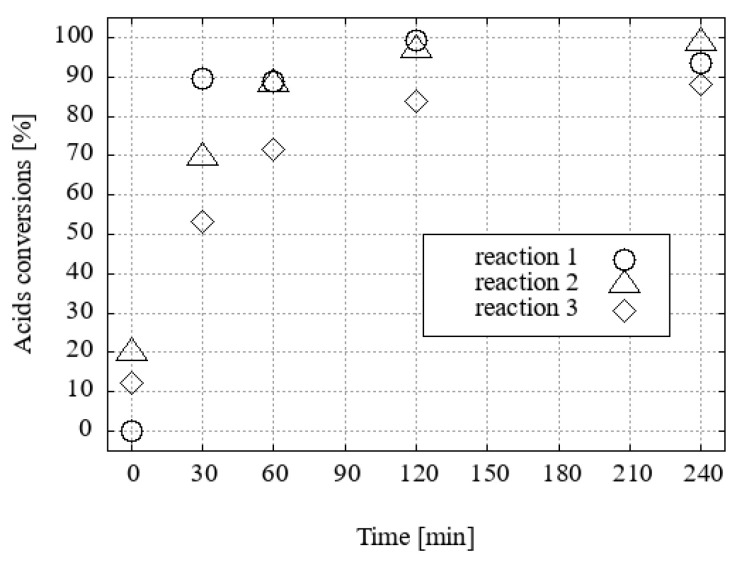
Repeatability of fatty acid conversions with methanol and 1 mol. % of H_3_O[Cl_2_-**1^−^**] after 3 reactions after reactivation of the catalyst on Amberlyst15.

**Figure 15:**
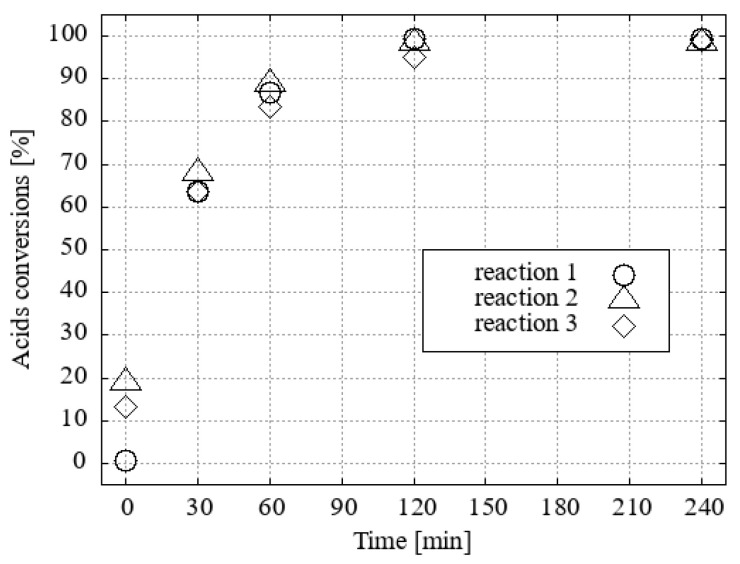
Repeatability of fatty acid conversions with ethanol and 1 mol. % of H_3_O[Cl_2_-**1^−^**] after 3 reactions after reactivation of the catalyst on Amberlyst15.

Consequently, following the completion of each reaction, a greater proportion of the catalyst is recovered in the form of its sodium salt rather than the acid. The salts of [Cl_2_-**1^−^**] were successfully regenerated to pure acid H_3_O[Cl_2_-**1^−^**] when passed through the CH_3_OH/HCl(aq)-activated ion exchanger Amberlyst15 (Figure 14 and Figure 15). The elevated amounts of esters detected at the outset of the second and third experiments, both in the absence and presence of H_3_O[Cl_2_-**1^−^**] regeneration (Figure 12 and Figure 14, respectively, and Figure 13 and Figure 15), can be attributed to residual esters remaining bound to the catalyst following the distillation of the product. The presence of these esters does not affect the course of the new reaction. Furthermore, the quantity of the esters in the reaction mixture is independent of their initial presence after only 30 min of reaction time.

In all instances of the repeated experiments, the same initial stack of new fatty acids was utilised in accordance with the molar ratios delineated in the esterification experiment settings. Under these reaction conditions, in all three successive reactions, conversions approaching the limit of 90% FAEEs were achieved after a mere 1 h of boiling in ethanol.

### 2.5. Comparison of Reactivity of Individual Fatty Acids

**Figure 16:**
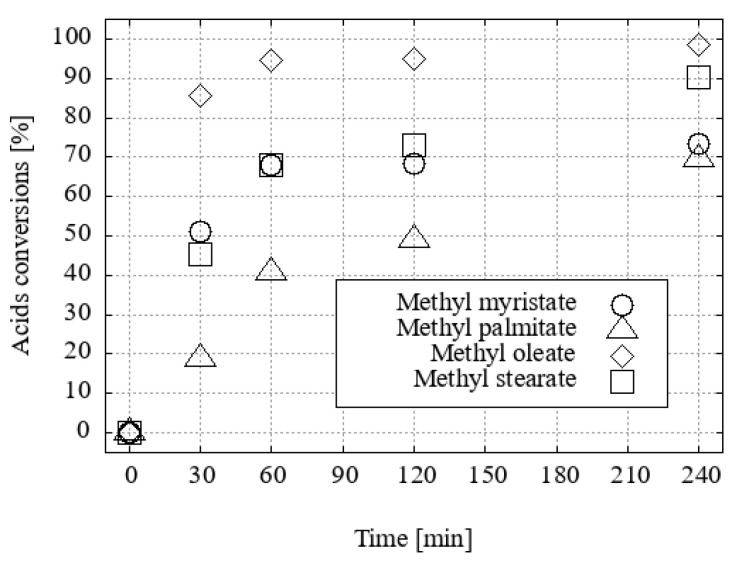
Differences in fatty acid conversions with 1 mol. % of H_3_O[Cl_2_-**1^−^**] in methanol.

**Figure 17:**
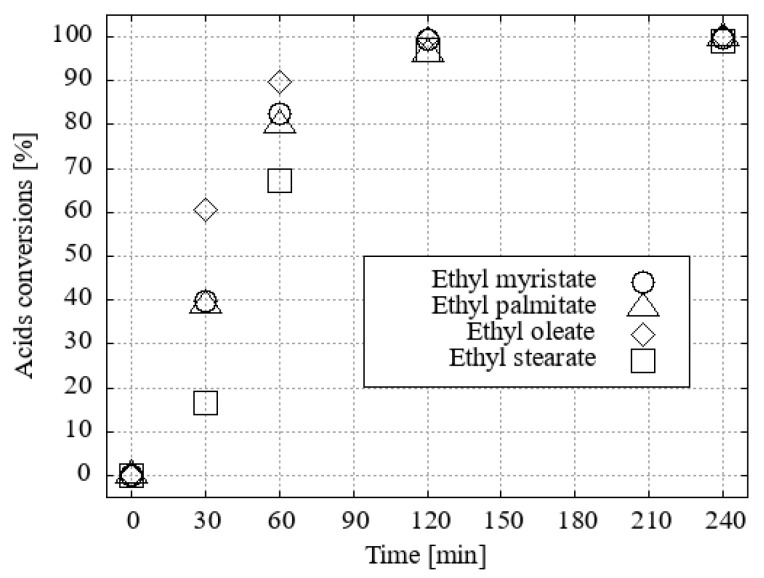
Differences in fatty acid conversions with 1 mol. % of H_3_O[Cl_2_-**1^−^**] in ethanol.

A mass range plotting script developed by Thermo Scientific Inc., Waltham, MA, USA was employed. Xcalibur v. 4.4.16.14 software was used to evaluate the conversions of each input fatty acid line to its methyl or ethyl ester. This was based on the knowledge of *m*/*z* reactants and products, as illustrated in Figure S15, derived from the measured HPLC APCI MS chromatograms. The fastest conversion was observed for oleic acid, both to methyl oleate (Figure 16) and ethyl oleate (Figure 17). In ethanol, the slowest conversion was observed for stearic acid to ethyl stearate. However, the conversions of each model ethyl ester reached more than 95% after just two hours of boiling in EtOH with 1 molar percent of H_3_O[Cl_2_-**1^−^**] as the homogeneous acid catalyst.

The conversion in methanol was observed to occur at a slower rate and with less uniformity. The stearic acid exhibited the least reactivity among the acids tested. While the conversion rate of oleic acid to methyl oleate exceeded 90% after 60 min, the conversion of stearic acid to methyl stearate required two hours to reach the same level of conversion, and both palmitic and myristic acid remained even after two hours, with a conversion of approximately 70%.

As anticipated, oleic acid, which is less symmetrical due to one double bond, reacts with the corresponding esters with the greatest preference. After half an hour of boiling, as little as 85% conversion was observed. Myristic acid and stearic acid also exhibit a similar trend of reaction, although the conversion of stearic acid occurs relatively rapidly. In contrast, the progression of palmitic acid is more gradual.

**Figure 18:**
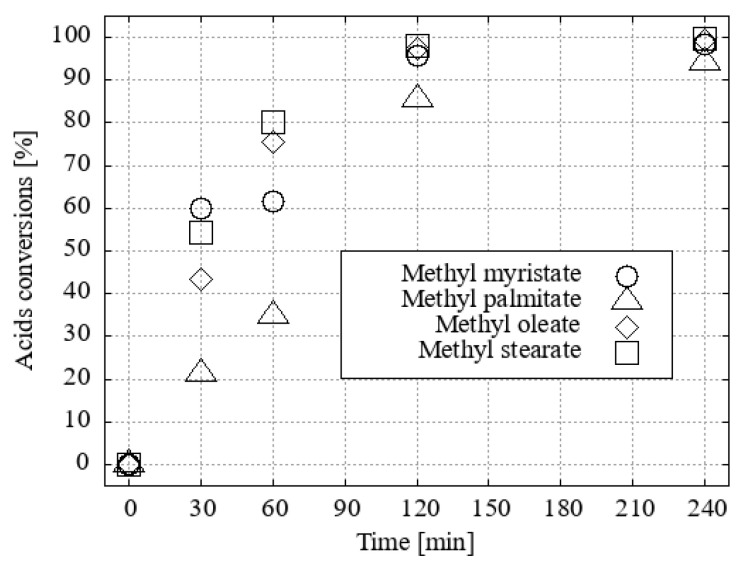
Differences in fatty acid conversions with 1 mol. % of H_3_O.HSO_4_ in methanol.

**Figure 19:**
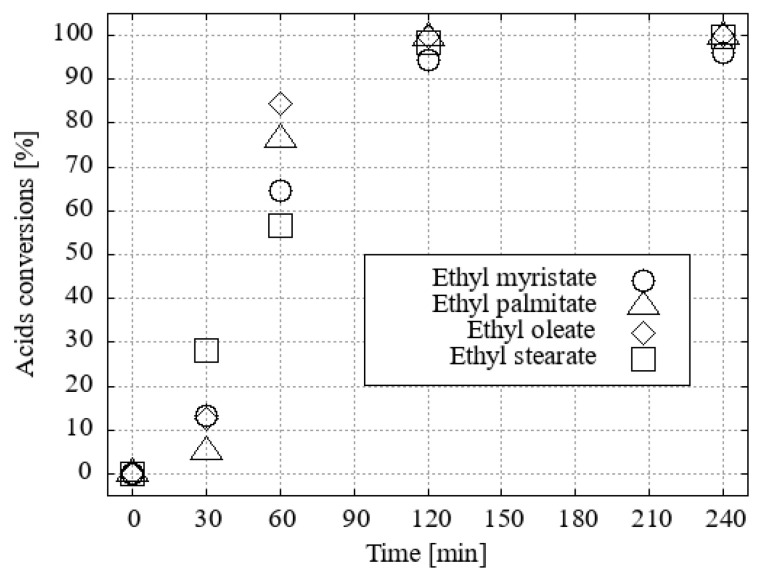
Differences in fatty acid conversions with 1 mol. % of H_3_O.HSO_4_ in ethanol.

The H_3_O[Cl_2_-**1^−^**] catalyst is generally comparable in efficiency to H_3_O[HSO_4_] according to the results of acid conversions (Figure 18 and Figure 19) and the TON/TOF values presented in Tables S3–S8. As demonstrated in Tables S3 and S5, H_3_O[Cl_2_-**1^−^**], in comparison to H_3_O[HSO_4_] (Tables S4 and S6), enables the conversion of 276.3 moles of free fatty acids to FAMEs and 297.2 moles to FAEEs within a 240 min period following three reactions with catalyst regeneration utilising an ion exchanger. The resulting TOF values demonstrate that one mole of fatty acids is converted with the assistance of a one-mole percent catalyst, H_3_O[Cl_2_-**1^−^**], after 240 min at boiling point with continuous drying of the reaction mixture at a frequency of 7.0 × 10^−3^ s^−1^.

## 3. Materials and Methods

### 3.1. Chemicals

The following chemical reagents, analytical standards, and highly pure organic solvents and materials were used in this study: COSAN.Cs (Katchem Praha CZ), *n-Bu*tyllithium 2.5M in Hexane (Lach-Ner CZ, 213351000), F-TEDA(BF_4_)_2_ 1-Chloromethyl-4-fluoro-1,4-diazonibicyclo[2.2.2]octane bis(tetrafluoroborate) (Sigma-Aldrich CZ, 439479), Hydrochloric acid 5N Biopharma (VWR CZ, 85400.320), Potasium permanganate (Lach-Ner CZ, 30123-AP0), Iodine (Lach-Ner CZ, 30111-AP0), Bromine 99.8% (VWR CZ, #00905.14), Sodium metal dry stick (VWR CZ, #ACRO3700212500), Benzophenone 99% (VWR CZ, #ACRO105565000), Diethylether (Lach-Ner CZ, 20018-ATO-M5000-1), Acetone HPLC grade (Lach-Ner CZ, A/0606/17), Acetonitrile LC-MS grade, Optima^®^ (Lach-Ner CZ, A955-212), Water LC-MS grade, Optima^®^ (Lach-Ner CZ, W6-212), Methanol UHPLC grade, Optima^®^ (Lach-Ner CZ, M/4070/PB17), Ethanol 96%, AnalaR Normapur (WVR CZ, 20823.327), Hexylamine 99% (Sigma-Aldrich CZ, 219703), Acetic acid glacial, >99.99% (Sigma-Aldrich CZ, 338826), Myristic acid (Sigma-Aldrich CZ, M3128) (Thermo Scientific CZ, 02744), Palmitic acid (Sigma-Aldrich CZ, P0500), Oleic acid, technical grade 90% (Sigma-Aldrich CZ, 364525), Stearic acid, reagent grade 95% (Sigma-Aldrich CZ, 175366), Ethyl stearate analytical standard (Sigma-Aldrich CZ, 18377), Ethyl oleate analytical standard (Sigma-Aldrich CZ, 55441), Ethyl palmitate analytical standard (Sigma-Aldrich CZ, 67107), Ethyl myristate 99% GC (Sigma-Aldrich CZ, 04597), Methyl stearate analytical standard (Sigma-Aldrich CZ, 85769), Methyl oleate (Sigma-Aldrich CZ, 268011, 98%), Methyl palmitate (Sigma-Aldrich CZ, P9009), Methyl myristate (Sigma-Aldrich, 70129), Amberlyst 15 (Sigma-Aldrich CZ, 06423), Molecular sieve type 3Å, technical, 1.6–2. 5 mm beads, and 8–12 mesh (Lach-Ner CZ, MS/1010/53). 1,2-dimethoxyethan 99+% (VWR CZ, ACRO118450025) was distilled from the dark blue Sodium ketyl complex solution under an argon atmosphere immediately prior to use.

### 3.2. UV-Vis Determination of Acidity Function H_0_

In order to ascertain *H*_0_ in accordance with Equation (2), the procedure proposed in [39] was employed, with Sudan Black B selected as the indicator (p*K*(IH^+^)_aq_ = 3.12) (Figure 5). The concentration ratios [I]/[IH^+^] were calculated from UV-Vis spectra in accordance with the Lambert–Beer law. The primary benefit of Sudan Black B is its absorption maximum at 596–605 nm, which is consistently outside the region of maximum absorption (240–430 nm) of the studied heteroboranoic acids (Figure 6). It is not possible to directly determine the value of p*K*(IH^+^)_aq_, as Sudan Black B is insoluble in water. However, titration in a water–ethanol mixture (1:1) was employed to ascertain its p*K*_a_, which was found to be 3.44 [98]. Brønsted–Lowry H_3_O^+^ acids were synthesised from the cesium salts of [**1^−^**], [F_2_-**1^−^**], [Cl_2_-**1^−^**], [Br_2_-**1^−^**], [I_1_-**1^−^**], [Cl_4_-**1^−^**], and [Br_4_-**1^−^**] (Figure 6) via the passage of substances dissolved in methanol through the activated ion exchanger Amberlyst 15. The evaporation of the sample on a vacuum rotary evaporator, followed by evaporation at an oil pump vacuum overnight at 50 °C (oil bath), yielded solid heteroboranoic acid. The acids were dissolved in methanol, with the concentration of water in the solution determined with the help of Karl Fischer titration with coulometry (WTD Coulometer, Diram Ltd., Prague, Czech Republic) to be 978.33 ± 132.54 μg H_2_O/g. This solution was then mixed with the dye indicator in a 75/25 (*v*/*v*) methanol–water mixture.

### 3.3. HPLC PDA MS

The HPLC separation method and data interpretation were guided by knowledge of the chromatographic behaviour of specific borane clusters [99]. The HPLC system Surveyor Plus (Thermo Scientific Inc., USA) [100] was employed, comprising a PDA Plus detector, Autosampler Plus, LC Pump Plus, and LCQ Fleet mass spectrometer equipped with an ESI probe. This was used for the detection and purity check of all heteroboranes prepared for this study, which were separated before detection on an ARION^®^ C8 250 × 3 mm, 5 µm HPLC column (Chromservis Ltd., Petrovice, Czech Republic). Through this column flowed an isocratic mobile phase comprising acetonitrile and water (60/40, *v*/*v*) at a flow rate of 0.75 cm^3^/min. The mobile phase also contained a 3 mM hexylamine formiate buffer at a pH of 4.99, in accordance with the recommendations set forth in [99]. The APCI ionisation probe was employed for the determination of the relative amounts of fatty acids in relation to FAMEs or FAEEs. Similarly, the stainless steel columns LUNA^®^ C8(2) 100Å, 250 × 3 mm, 5 µm (Phenomenex Inc., Torrance, CA, USA), or Purospher^®^ LiChroCART^®^ STAR RP8e, 250 × 2 mm, 5 µm (Merck, Darmstadt, Germany) were utilised. In order to achieve simultaneous analytical separation of fatty acids, FAMEs, and FAEEs, a gradient mobile phase with a flow rate of 0.3 cm^3^/min was employed. This began with an acetonitrile and water (6/4, *v*/*v*) solution of hexylamine formiate buffer (pH = 4.99) at a concentration of 3 mM.

### 3.4. XRD

The single-crystal X-ray data were collected at 180 (2) K on a Bruker D8 Venture diffractometer equipped with Photon 100, Incoatec microfocus sealed tube Cu-Kα radiation. The data were integrated, scaled, and corrected for absorption using Apex3 [101]. The structure was solved with the help of SUPERFLIP [102] and anisotropically refined using full matrix least squares on *F* squared using the CRYSTALS [103] to the final value of *R* = 0.045 and *wR* = 0.099 using 3042 independent reflections (*θ_max_* = 70.06°), 201 parameters, and 1 restraint. The hydrogen atoms bonded to boron and carbon atoms were placed in calculated positions and refined with riding constraints. The disordered functional groups’ positions were found in different electron density maps and refined with restrained geometry. MCE [104] was used for the visualisation of electron density maps. The occupancy of disordered chlorine atoms was refined. To stabilise the refinement, the occupancy values close to 0.5 were fixed at 0.5. The occupancy of [Cl_5_-**1^−^**] isomers, found to cocrystallise with [Cl_4_-**1^−^**], was fixed at 0.06.

### 3.5. NMR

JNM-ECZ400R/M1 with JASTEC 400/54/JJYH/W (9.39 T, 54 mm standard bore) (JEOL Ltd., Tokyo, Japan) operating at a resonance frequency of 400 MHz for the ^1^H, 100 MHz for the ^13^C ((CH_3_)_4_Si external reference for both nuclei, ^1^H and ^13^C), 128 MHz for ^11^B (BF_3_.O(C_2_H_5_)_2_ external reference), and 106 MHz for ^23^Na (NaCl as the external reference) nuclei was used for structural characterisation of mono- and dihalogenated derivatives of [**1^−^**], and especially for the determination of the ^23^Na content in methanol after time-dependent contact with 3Å molecular sieves. The data were processed with Delta JEOL [105] version 5.3.2. Both new sibling mixtures of [Cl_4_-**1^−^**] and [Br_4_-**1^−^**] were measured with a higher resolution of overlayed signals on a JEOL 600 MHz NMR spectrometer JNM-ECZ600R/M3 with a superconducting magnet JMTC-600/54J (14.09637 T, 54 mm standard bore).

### 3.6. Elemental CHN Analysis

Elemental analyses of C and H present in prepared dry Cs salts of [**1^−^**], [F_2_-**1^−^**], [Cl_2_-**1^−^**], [Br_2_-**1^−^**], [I_1_-**1^−^**], [Cl_4_-**1^−^**], and [Br_4_-**1^−^**] was performed using a Flash*Smart* analyser (Thermo Scientific Inc., USA) according to the manufacturer’s procedure for the determination of combustible compounds using the GC method. The reactor temperature was set at 950 °C, and the chromatography column was tempered to 75 °C. The carrier gas (He) flow rate was set at 140 cm^3^.min^−1^, the reference flow rate at 100 cm^3^.min^−1^ and the oxygen flow rate was 250 cm^3^.min^−1^ with the end of injection after 4 s. The sampling delay was set at 12 s, and the total analysis time was 600 s. Each sample was measured in duplicate, and acetanilide was used as the analytical standard. The data were evaluated in Eager Smart [106] software (version 1.00). The average of two measurements with relative standard deviation (RSD) is shown in the Supplementary Information (Table S1).

### 3.7. Melting Point

The determination of the melting points of the studied substances, which were enclosed in glass capillaries, was based on the performance of three repeated measurements on a Stuart SMP 10 spot meter. The initial temperature was set to 250 °C, and the melting temperature interval was read at the temperatures at which the crystals of the sample began to melt and at which the sample was completely liquefied.

## 4. Conclusions

The parent cobalt bis-dicarbollide acid, H_3_O[**1^−^**], and the halogen derivatives H_3_O[F_2_-**1^−^**], H_3_O[Cl_2_-**1^−^**], H_3_O[Br_2_-**1^−^**], H_3_O[I_1_-**1^−^**], H_3_O[Br_4_-**1^−^**], and H_3_O[Cl_4_-**1^−^**] were prepared and characterised to evaluate their acidity and catalytic activity in esterification of four fatty acids with methanol or ethanol. The halogen derivatives were synthesised via an unconventional methodology. A single crystal containing an equimolar mixture of the previously unreported isomer [8,8′,12,12′-Cl_4_-**1^−^**], a sibling to the already known [8,8′,9,9′-Cl_4_-**1^−^**], was obtained under strongly basic, anhydrous conditions [67]. Two further novel compounds, [8,8′,9,9′-Br_4_-**1^−^**] and [8,8′,12,12′-Br_4_-**1^−^**], were also obtained in an isomeric mixture. The structures of the prepared compounds were elucidated through the application of XRD, NMR, HPLC, and MS methodologies. In particular, the purity was verified through elemental CHN analysis and HPLC-MS. Heteroborane acids are orange hygroscopic solids with poor solubility in water but high solubility in methanol or methanol/water (75/25, *v*/*v*). It was thus necessary to identify an appropriate hydrophobic colour indicator for the determination of the acidity function. The use of Sudan Black B (III) (Figure 5) for UV-Vis determination of the Hammett acidity function of hydrophobic acids was found to be successful. The Hammett acidity of these heteroborane acids is remarkably similar to that of H_2_SO_4_. H_3_O[Cl_2_-**1^−^**] was identified as the most potent and stable acid within the series. The most effective conversion was achieved following a single reaction with 1 mol% of catalyst, with FAMEs reaching 98 wt.% of free fatty acids within one hour and FAEEs reaching 99 wt.% of FFAs within two hours. The issue of decreased efficiency with the catalyst upon repeated use, attributed to the exchange of H_3_O^+^ ions for Na^+^ ions, was identified with the help of ^23^Na NMR analysis. The catalyst [Cl_2_-**1^−^**] was successfully regenerated to a pure acid, H_3_O[Cl_2_-**1^−^**], through passage through a CH_3_OH/HCl-activated ion exchanger, Amberlyst15.

The utilisation of 3Å zeolite molecular sieves as a desiccant, situated above the reactor in the water vapour absorber, enables the attainment of exemplary yields of FAEEs, the reuse of the desiccant on numerous occasions, the circumvention of an increase in the excess volume of alcohol, and is thus an optimal choice for the prospective continuation of the process through the employment of a cascade of absorbers. The two-segment APCI +/− HPLC MS method revealed that oleic acid was the fastest reactant in the mixture of fatty acids tested. Consequently, only small amounts of saturated fatty acids remain in the distillation residue. Pretreatment of substrates by drying and desalting with an ion exchanger seems essential for the repeated use of these catalysts. The inhibition of the catalyst due to Na^+^ ions extracted from zeolite during the reaction suggests that the replacement of the zeolite with another efficient, cost-effective and easily regenerated desiccant or separation method could enhance the process. The most stable, hydrophobic, and hygroscopic, yet strongly gentle and acidic non-oxidising homogeneous catalyst capable of converting more than 97% of the studied mixture of four free fatty acids to FAMEs after 60 min and to FAEEs after 120 min was found to be 1 mol.% of H_3_O[Cl_2_-**1^−^**]. The H_3_O[Cl_2_-**1^-^**] catalyst was recycled three times with a total decrease of only 2% in the conversion of fatty acids into FAEEs.

## Figures and Tables

**Table 1:** The differences in *H*_0_, calculated from the UV-Vis absorption maxima of Sudan black B dye interacting with the studied acids, were observed when the acids were dissolved together in methanol (M) or in a methanol–water (75/25, *v*/*v*) mixture (MW).

Compound	λ_max_ [nm] Acid M	λ_max_ [nm] Dye M	λ_max_ [nm] Dye MW	*H°* M	*H°* MW	Δ*H°* M-MW
Sudan B dye	592	591	591	-	-	-
H_3_O^+^HSO_4_^−^	201	639	617	3.89	4.04	−0.15
H_3_O[**1^−^**]	297	631	600	3.93	4.11	−0.18
H_3_O[F_2_-**1^−^**]	301	634	608	4.40	4.16	+0.24
H_3_O[Cl_2_-**1^−^**]	319	634	628	4.11	3.88	+0.23
H_3_O[Cl_4_-**1^−^**]	320	639	608	3.95	4.18	−0.23
H_3_O[Br_2_-**1^−^**]	321	631	617	4.35	4.12	+0.23
H_3_O[Br_4_-**1^−^**]	325	639	617	3.95	4.26	−0.30
H_3_O[I_1_-**1^−^**]	314	639	609	4.52	4.08	+0.44

**Table 2:** ^23^Na NMR chemical shift peak area integral as proof of sodium cations extracted from the 3Å molecular sieve using hot methanol (M) or ethanol (E) under argon atmosphere after 0, 1, and 4 h of the reflux.

Alcohol	Time [h]	pos. X [ppm]	Integral [mabn]	Normalized Integral
MeOH	0	−3.256	5.95	5.95 × 10^−3^
MeOH	1	−3.179	15.30	1.53 × 10^−2^
MeOH	4	−3.133	40.91	4.09 × 10^−2^
EtOH	0	−0.107	0.69	6.91 × 10^−4^
EtOH	4	−0.76	2.90	2.89 × 10^−3^

## Data Availability

The data presented in this study are available at https://zenodo.org/records/14035885 (accessed on 4 November 2024).

## Supplementary Materials

The following supporting information can be downloaded at https://www.mdpi.com/article/10.3390/ijms252413263/s1. CSD 2375647 contains supplementary crystallographic data for this paper. These data can be obtained free of charge via https://www.ccdc.cam.ac.uk/structures/, or by e-mailing data_request@ccdc.cam.ac.uk, or by contacting The Cambridge Crystallographic Data Centre, 12 Union Road, Cambridge CB2 1EZ, UK.

## Appendix A

**Table A1:** Comparison of H_3_O[Cl_2_-**1^−^**] and H_2_SO_4_ as homogeneous catalysts used with methanol for esterification to FAMEs at various experimental conditions.

Catalyst	Mol%	Wt.%	Methanol/Oil *w*/*w* Ratio (Mol Ratio)	Temp. [°C]	Time [h]	Acids Conversion [%]	Drying	Citation	Substrate
No	0	0	4:1 (25:1)	100	4		Zeolite 3Å	This work	Mixture of myristic, palmitic, stearic, oleic acids
Cl_2_-[**1^−^**]	1.0	1.5	4:1 (25:1)	100	4	85.1	Not used	This work	
Cl_2_-[**1^−^**]	1.0	1.5	4:1 (25:1)	100	1	96.7	Zeolite 3Å	This work	
H_2_SO_4_	1.0	1.5	4:1 (25:1)	100	2	98.1	Zeolite 3Å	This work	
H_2_SO_4_	5.0	1.9	30:1	35	0.16	99.9	Predistillation	[11]	Soapstock
H_2_SO_4_		5.0	20:1	60	0.5	91.73	Not used	[25]	Soybean oil
H_2_SO_4_		5	30:1	35	0.16	99	Freeze-drying with a falling film evaporator	[15]	Soybean oil
H_2_SO_4_		3.8	245:1	80	4	99.1	Not used	[12]	Waste cooking oil (WCO)
H_2_SO_4_		0.1	(3:1)	130	0.5	95	Dry methanol at start	[17]	Palm fatty acids
H_2_SO_4_		1.25- 2.5	30:1	50	24	99.0 - 93.2	Not used	[14]	Tallow
H_2_SO_4_		1.0	3:1	100	1.3	88.0	Not used	[19]	Oleic acid
H_2_SO_4_		1.0	3:1	100	1.3	97.2	Zeolite 3Å	[19]	Oleic acid
H_2_SO_4_		1.0	9:1	100	1	99.0	Zeolite 3Å	[22]	Coconut oil (98% FFA)
H_2_SO_4_		0.5	6:1	65	4	99.9	Not used	[2]	Animal fats
H_2_SO_4_		5.0	30:1	65	3	93.24	Not used	[3]	WCO
H_2_SO_4_		?	2:1	100	1	69	Zeolite 3Å	[21]	20% oleic acid added to soybean oil
H_2_SO_4_		5.4	30% MeOH	50	2	<97	175 wt.% of dry MgSO_4_	[1]	Fat Trap Grease
H_2_SO_4_		7.4	5:1	60	3	99.34		[4]	PFAD
H_2_SO_4_		25.0	9:1	65	2	99.08	Not used	[26]	PFAD
H_2_SO_4_		0.5	15:1	55	1	96.8	Not used	[27]	Tall oil
H_2_SO_4_		8.0	8:1	50	4	96.5	Not used	[28]	FOG
H_2_SO_4_		0.05 g/g FFA	2.5 g MeOH/g FFA	55 ± 2	0.5	95.5	10 min oil preheating—H_2_O below 0.1%	[16]	Rubber seed oil

**Table A2:** Comparison of H_3_O[Cl_2_-**1^−^**] and H_2_SO_4_ as homogeneous catalysts used with ethanol for esterification to FAEEs at various experimental conditions.

Catal.	Mol%	Wt.%	Ethanol/Oil *w/w* Ratio (Mol Ratio)	Temp [°C]	Time [h]	Acids Conversion [%]	Drying	Citation	Substrate
No	0.0	0.0	4:1 (25:1)	100	4	0.0	Not used	This work	Mixture of myristic, palmitic, stearic, oleic acids
Cl_2_-[**1^−^**]	1.0	1.5	4:1 (25:1)	100	1	96.7	Zeolite 3Å	This work	
Cl_2_-[**1^−^**]	1.0	1.5	4:1 (25:1)	100	4	85.1	Not used	This work	
H_2_SO_4_	1.0	1.5	4:1 (25:1)	100	2	98.9	Zeolite 3Å	This work	
H_2_SO_4_		0.1	(3:1)	130	1	85	Dry ethanol at start	[17]	Palm FFA
H_2_SO_4_		2.3	6:1	55	2	>93	Not used	[10]	Sunflower oil + oleic acid
H_2_SO_4_		5.0	3:1	60	2	>90	Not used	[29]	Oleic acid
H_2_SO_4_		1.0	3:1	100	1	99.9	Zeolite 3Å	[20]	Oleic acid
H_2_SO_4_		1.0	9:1	100	1	99.0	Zeolite 3Å	[22]	Coconut oil (98% FFA)
H_2_SO_4_		1.0	15:1	70	2	99	Dry ethanol	[18]	18% FFA, 82% sunflower oil
H_2_SO_4_		?	3:1	100	1	69	Zeolite 3Å	[21]	20% oleic acid added to soybean oil
H_2_SO_4_		2.1	57 vol%	50				[30]	PFAD, ultrasound, continuous

## References

[1] Kadi M.E.A., Awad S., Loubar K., Akkouche N., Tazerout M. (2020). Experimental Study on the Esterification of Fat Trap Grease in a Continuous Reactor. Waste Biomass Valor..

[2] Encinar J.M., Sánchez N., Martínez G., García L. (2011). Study of Biodiesel Production from Animal Fats with High Free Fatty Acid Content. Bioresour. Technol..

[3] Li J., Zhou H.B., Cao Y. (2012). Transesterification of Waste Cooking Oil to Produce Biodiesel Using Acid and Alkaline Catalyst. AMR.

[4] Ulfah M., Firdaus, Sundari E., Praputri E. (2020). A Comparison of Palm Fatty Acid Distillate (PFAD) Esterification Using Sulphated Alumina versus Sulphuric Acid Catalyst. IOP Conf. Ser. Mater. Sci. Eng..

[5] Zhou W., Konar S.K., Boocock D.G.B. (2003). Ethyl Esters from the Single-phase Base-catalyzed Ethanolysis of Vegetable Oils. J. Am. Oil Chem. Soc..

[6] Ji J., Wang J., Li Y., Yu Y., Xu Z. (2006). Preparation of Biodiesel with the Help of Ultrasonic and Hydrodynamic Cavitation. Ultrasonics.

[7] Šánek L., Pecha J., Kolomazník K., Bařinová M. (2016). Pilot-Scale Production of Biodiesel from Waste Fats and Oils Using Tetramethylammonium Hydroxide. Waste Manag..

[8] KoohiKamali S., Tan C.P., Ling T.C. (2012). Optimization of Sunflower Oil Transesterification Process Using Sodium Methoxide. Sci. World J..

[9] Fischer E., Speier A. (1895). Darstellung Der Ester. Ber. Dtsch. Chem. Ges..

[10] Marchetti J.M., Errazu A.F. (2008). Esterification of Free Fatty Acids Using Sulfuric Acid as Catalyst in the Presence of Triglycerides. Biomass Bioenergy.

[11] Haas M.J., Bloomer S., Scott K. (2000). Simple, High-efficiency Synthesis of Fatty Acid Methyl Esters from Soapstock. J. Am. Oil Chem. Soc..

[12] Zheng S., Kates M., Dubé M.A., McLean D.D. (2006). Acid-Catalyzed Production of Biodiesel from Waste Frying Oil. Biomass Bioenergy.

[13] Haas M.J., Michalski P.J., Runyon S., Nunez A., Scott K.M. (2003). Production of FAME from Acid Oil, a By-product of Vegetable Oil Refining. J. Am. Oil Chem. Soc..

[14] Bhatti H., Hanif M., Qasim M. (2008). Ataurrehman Biodiesel Production from Waste Tallow. Fuel.

[15] Haas M.J. (2005). Improving the Economics of Biodiesel Production through the Use of Low Value Lipids as Feedstocks: Vegetable Oil Soapstock. Fuel Process. Technol..

[16] Gunawardena S., Walpita D.H., Ismail M. (2017). Method for Quantification of Methanol and Sulfuric Acid Required for Esterification of High Free Fatty Acid Oils in Biodiesel Production. Int. J. Renew. Energy Res..

[17] Aranda D.A.G., Santos R.T.P., Tapanes N.C.O., Ramos A.L.D., Antunes O.A.C. (2008). Acid-Catalyzed Homogeneous Esterification Reaction for Biodiesel Production from Palm Fatty Acids. Catal. Lett..

[18] Pisarello M.L., Dalla Costa B., Mendow G., Querini C.A. (2010). Esterification with Ethanol to Produce Biodiesel from High Acidity Raw Materials. Fuel Process. Technol..

[19] Lucena I.L., Silva G.F., Fernandes F.A.N. (2008). Biodiesel Production by Esterification of Oleic Acid with Methanol Using a Water Adsorption Apparatus. Ind. Eng. Chem. Res..

[20] Lucena I.L., Saboya R.M.A., Oliveira J.F.G., Rodrigues M.L., Torres A.E.B., Cavalcante C.L., Parente E.J.S., Silva G.F., Fernandes F.A.N. (2011). Oleic Acid Esterification with Ethanol under Continuous Water Removal Conditions. Fuel.

[21] Touma J.G., El Khoury B., Estephane J., Zakhem H., Aouad S. (2018). Effect of Alcohol Type and Amount on the Total Energy Consumption and Yield of the Free Fatty Acids Esterification Reaction with Simultaneous Adsorptive Water Removal. Chem. Eng. Commun..

[22] Oliveira J.F.G., Lucena I.L., Saboya R.M.A., Rodrigues M.L., Torres A.E.B., Fernandes F.A.N., Cavalcante C.L., Parente E.J.S. (2010). Biodiesel Production from Waste Coconut Oil by Esterification with Ethanol: The Effect of Water Removal by Adsorption. Renew. Energy.

[23] Jamil F., Saleem M., Ali Qamar O., Khurram M.S., Al-Muhtaseb A.H., Inayat A., Akhter P., Hussain M., Rafiq S., Yim H. (2023). State-of-the-Art Catalysts for Clean Fuel (Methyl Esters) Production—a Comprehensive Review. J. Phys. Energy.

[24] Khan Z., Javed F., Shamair Z., Hafeez A., Fazal T., Aslam A., Zimmerman W.B., Rehman F. (2021). Current Developments in Esterification Reaction: A Review on Process and Parameters. J. Ind. Eng. Chem..

[25] Canakci M., Van Gerpen J. (2001). Biodiesel Production from Oils and Fats with High Free Fatty Acids. Trans. ASAE.

[26] Tambun R., Burmana A.D., Alexander V. (2022). The Effect of Reactant Molar Ratio to Performance of H_2_SO_4_ Catalyst and para-Toluene Sulfonic Acid Catalyst in The Esterification of Palm Fatty Acid Distillate into Biodiesel. J. Eng. Sci. Technol..

[27] Lawer-Yolar G., Dawson-Andoh B., Atta-Obeng E. (2021). Synthesis of Biodiesel from Tall Oil Fatty Acids by Homogeneous and Heterogeneous Catalysis. Sustain. Chem..

[28] Utlang S.M.S., Utlang N.M.S., Paler E.M.L., Salvatierra R.C., Paday J.C., Mugot D.A., Mabayo V.I.F., Arazo R.O. (2023). Optimized Recovery of Transesterifiable Oil from Industrial Fats, Oil, and Grease (FOG) Esterified with H2SO4 Catalyst Extracted from Discarded Lead-Acid Batteries. Energ. Ecol. Environ..

[29] Hanh H.D., Dong N.T., Okitsu K., Nishimura R., Maeda Y. (2009). Biodiesel Production by Esterification of Oleic Acid with Short-Chain Alcohols under Ultrasonic Irradiation Condition. Renew. Energy.

[30] Somnuk K., Phanyusoh D., Thawornprasert J., Oo Y.M., Prateepchaikul G. (2021). Continuous Ultrasound-Assisted Esterification and Transesterification of Palm Fatty Acid Distillate for Ethyl Ester Production. Processes.

[31] Trombettoni V., Lanari D., Prinsen P., Luque R., Marrocchi A., Vaccaro L. (2018). Recent Advances in Sulfonated Resin Catalysts for Efficient Biodiesel and Bio-Derived Additives Production. Prog. Energy Combust. Sci..

[32] Lee D.-W., Lee K.-Y. (2014). Heterogeneous Solid Acid Catalysts for Esterification of Free Fatty Acids. Catal. Surv. Asia.

[33] Rizwanul Fattah I.M., Ong H.C., Mahlia T.M.I., Mofijur M., Silitonga A.S., Rahman S.M.A., Ahmad A. (2020). State of the Art of Catalysts for Biodiesel Production. Front. Energy Res..

[34] Atabani A.E., Silitonga A.S., Badruddin I.A., Mahlia T.M.I., Masjuki H.H., Mekhilef S. (2012). A Comprehensive Review on Biodiesel as an Alternative Energy Resource and Its Characteristics. Renew. Sustain. Energy Rev..

[35] Maheshwari P., Haider M.B., Yusuf M., Klemeš J.J., Bokhari A., Beg M., Al-Othman A., Kumar R., Jaiswal A.K. (2022). A Review on Latest Trends in Cleaner Biodiesel Production: Role of Feedstock, Production Methods, and Catalysts. J. Clean. Prod..

[36] Troter D.Z., Todorović Z.B., Đokić-Stojanović D.R., Stamenković O.S., Veljković V.B. (2016). Application of Ionic Liquids and Deep Eutectic Solvents in Biodiesel Production: A Review. Renew. Sustain. Energy Rev..

[37] Mardhiah H.H., Ong H.C., Masjuki H.H., Lim S., Lee H.V. (2017). A Review on Latest Developments and Future Prospects of Heterogeneous Catalyst in Biodiesel Production from Non-Edible Oils. Renew. Sustain. Energy Rev..

[38] Sekoai P.T., Ouma C.N.M., Du Preez S.P., Modisha P., Engelbrecht N., Bessarabov D.G., Ghimire A. (2019). Application of Nanoparticles in Biofuels: An Overview. Fuel.

[39] Hammett L.P., Deyrup A.J. (1932). A Series of Simple Basic Indicators. I. The Acidity Functions of Mixtures of Sulfuric and Perchloric Acids with Water. J. Am. Chem. Soc..

[40] Gillespie R.J. (1968). Fluorosulfuric Acid and Related Superacid Media. Acc. Chem. Res..

[41] Gillespie R.J., Liang J. (1988). Superacid Solutions in Hydrogen Fluoride. J. Am. Chem. Soc..

[42] Fărcasiu D., Hâncu D. (1997). Acid Strength of Tetrafluoroboric Acid The Hydronium Ion as a Superacid and the Inapplicability of Water as an Indicator of Acid Strength. J. Chem. Soc. Faraday Trans..

[43] Juhasz M., Hoffmann S., Stoyanov E., Kim K., Reed C.A. (2004). The Strongest Isolable Acid. Angew Chem Int Ed.

[44] Gräsvik J., Hallett J.P., To T.Q., Welton T. (2014). A Quick, Simple, Robust Method to Measure the Acidity of Ionic Liquids. Chem. Commun..

[45] Himmel D., Goll S.K., Leito I., Krossing I. (2010). A Unified pH Scale for All Phases. Angew. Chem. Int. Ed..

[46] Mihichuk L.M., Driver G.W., Johnson K.E. (2011). Brønsted Acidity and the Medium: Fundamentals with a Focus on Ionic Liquids. ChemPhysChem.

[47] Panchal B., Bian K., Chang T., Zhu Z., Wang J., Qin S., Zhao C., Sun Y. (2021). Synthesis of Generation-2 Polyamidoamine Based Ionic Liquid: Efficient Dendrimer Based Catalytic Green Fuel Production from Yellow Grease. Energy.

[48] He L., Qin S., Chang T., Sun Y., Gao X. (2013). Biodiesel Synthesis from the Esterification of Free Fatty Acids and Alcohol Catalyzed by Long-Chain Brønsted Acid Ionic Liquid. Catal. Sci. Technol..

[49] Thomazeau C., Olivier-Bourbigou H., Magna L., Luts S., Gilbert B. (2003). Determination of an Acidic Scale in Room Temperature Ionic Liquids. J. Am. Chem. Soc..

[50] Reed C.A., Kim K.-C., Bolskar R.D., Mueller L.J. (2000). Taming Superacids: Stabilization of the Fullerene Cations HC_60_^+^ and C_60_^+^. Science.

[51] Reed C.A. (2005). Carborane Acids. New “Strong yet Gentle” Acids for Organic and Inorganic Chemistry. Chem. Commun..

[52] Cummings S., Hratchian H.P., Reed C.A. (2016). The Strongest Acid: Protonation of Carbon Dioxide. Angew. Chem. Int. Ed..

[53] Stoyanov E.S., Stoyanova I.V. (2018). Features of Protonation of the Simplest Weakly Basic Molecules, SO_2_, CO, N_2_O, CO_2_, and Others by Solid Carborane Superacids. Angew. Chem. Int. Ed..

[54] Hawthorne M.F., Andrews T.D. (1965). Carborane Analogues of Cobalticinium Ion. Chem. Commun..

[55] Verdiá-Báguena C., Alcaraz A., Aguilella V.M., Cioran A.M., Tachikawa S., Nakamura H., Teixidor F., Viñas C. (2014). Amphiphilic COSAN and I2-COSAN Crossing Synthetic Lipid Membranes: Planar Bilayers and Liposomes. Chem. Commun..

[56] Strauss S.H. (1993). The Search for Larger and More Weakly Coordinating Anions. Chem. Rev..

[57] Reed C.A. (1998). Carboranes: A New Class of Weakly Coordinating Anions for Strong Electrophiles, Oxidants, and Superacids. Acc. Chem. Res..

[58] Xie Z., Jelinek T., Bau R., Reed C.A. (1994). New Weakly Coordinating Anions. III. Useful Silver and Trityl Salt Reagents of Carborane Anions. J. Am. Chem. Soc..

[59] Plešek J., Baše K., Mareš F., Hanousek F., Štíbr B., Heřmánek S. (1984). Potential Uses of Metallocarborane Sandwich Anions for Analysis, Characterization and Isolation of Various Cations and Organic Bases. Collect. Czech. Chem. Commun..

[60] Rais J., Plešek J., Selucký P., Kyrš M., Kadlecová L. (1991). Extraction of Cesium with Derivatives of Carborane into Nitrobenzene. J. Radioanal. Nucl. Chem..

[61] Fanning J.C., Huff L.A., Smith W.A., Terrell A.S., Yasinsac L., Todd L.J., Jasper S.A., McCabe D.J. (1995). Caesium Cobalt Dicarbollide—Solubility, Precipitation and Reactivity in Basic Aqueous Solution. Polyhedron.

[62] Bühl M., Hnyk D., Macháček J. (2005). Computational Study of Structures and Properties of Metallaboranes: Cobalt Bis(Dicarbollide). Chem. A Eur. J..

[63] Hawthorne M.F., Young D.C., Andrews T.D., Howe D.V., Pilling R.L., Pitts A.D., Reintjes M., Warren L.F., Wegner P.A. (1968). .pi.-Dicarbollyl Derivatives of the Transition Metals. Metallocene Analogs. J. Am. Chem. Soc..

[64] Rais J., Selucký P., Kyrš M. (1976). Extraction of Alkali Metals into Nitrobenzene in the Presence of Univalent Polyhedral Borate Anions. J. Inorg. Nucl. Chem..

[65] Sengupta A.K., Marcus Y. (2004). Ion Exchange and Solvent Extraction: A Series of Advances.

[66] Schulz W.W., Bray L.A. (1987). Solvent Extraction Recovery of Byproduct ^137^Cs and ^90^Sr from HNO_3_ Solutions—A Technology Review and Assessment. Sep. Sci. Technol..

[67] Mátel Ľ., Macášek F., Rajec P., Heřmánek S., Plešek J. (1982). B-Halogen Derivatives of the Bis(1,2-Dicarbollyl)Cobalt(III) Anion. Polyhedron.

[68] Hurlburt P.K., Miller R.L., Abney K.D., Foreman T.M., Butcher R.J., Kinkhead S.A. (1995). New Synthetic Routes to B-Halogenated Derivatives of Cobalt Dicarbollide. Inorg. Chem..

[69] Rudakov D.A., Shirokii V.L., Knizhnikov V.A., Bazhanov A.V., Vecher E.I., Maier N.A., Potkin V.I., Ryabtsev A.N., Petrovskii P.V., Sivaev I.B. (2004). Electrochemical Synthesis of Halogen Derivatives of Bis(1,2-Dicarbollyl)Cobalt(III). Russ. Chem. Bull..

[70] Farràs P., Viñas C., Teixidor F. (2013). Preferential Chlorination Vertices in Cobaltabisdicarbollide Anions. Substitution Rate Correlation with Site Charges Computed by the Two Atoms Natural Population Analysis Method (2a-NPA). J. Organomet. Chem..

[71] Koval M., Kaniansky D., Mátel L., Macáśek F. (1982). Analysis of Boron–Halogen Derivatives of Bis(1,2-Dicarbollyl)Cobalt(III) Anions by Capillary Isotachophoresis. J. Chromatogr. A.

[72] Šimuničová E., Kaniansky D. (1987). Isotachophoretic Determination of Chloro Derivatives of Cobaltocarborane without the Use of Reference Analytes. J. Chromatogr. A.

[73] Sivý P., Preisinger A., Baumgartner O., Valach F., Koreň B., Mátel L. (1986). Structure of Caesium 3,3’-Commo-Bis(8,9,12-Tribromooctahydro-1,2-Dicarba-3-Cobalta-Closo-Dodecaborate)(1-). Acta Crystallogr. C Cryst. Struct. Commun..

[74] Ruhle H.W., Hawthorne M.F. (1968). .pi.-Dicarbollyl Derivatives of Chromium. Metallocene Analogs. Inorg. Chem..

[75] Kazheva O.N., Aleksandrov G.G., Kravchenko A.V., Starodub V.A., Zhigareva G.G., Sivaev I.B., Bregadze V.I., Buravov L.I., Titov L.V., D’yachenko O.A. (2010). Synthesis, Structures, and Conductivities of Salts (BEDT-TTF)[9,9′(12′)-I_2_-3,3′-Co(1,2-C_2_B_9_H_10_)_2_] and (TTF)[9,9′,12,12′-I_4_-3,3′-Co(1,2-C_2_B_9_H_9_)_2_]. Russ. Chem. Bull..

[76] Anufriev S.A., Sivaev I.B., Bregadze V.I. (2015). Synthesis of 9,9´,12,12´-Substituted Cobalt Bis(Dicarbollide) Derivatives. Russ. Chem. Bull..

[77] El Anwar S., Růžičková Z., Bavol D., Fojt L., Grüner B. (2020). Tetrazole Ring Substitution at Carbon and Boron Sites of the Cobalt Bis(Dicarbollide) Ion Available via Dipolar Cycloadditions. Inorg. Chem..

[78] Chamberlin R.M., Scott B.L., Melo M.M., Abney K.D. (1997). Butyllithium Deprotonation vs Alkali Metal Reduction of Cobalt Dicarbollide: A New Synthetic Route to C-Substituted Derivatives. Inorg. Chem..

[79] Fino S.A., Benwitz K.A., Sullivan K.M., LaMar D.L., Stroup K.M., Giles S.M., Balaich G.J., Chamberlin R.M., Abney K.D. (1997). Condensation Polymerization of Cobalt Dicarbollide Dicarboxylic Acid. Inorg. Chem..

[80] Grüner B., Švec P., Šícha V., Padělková Z. (2012). Direct and Facile Synthesis of Carbon Substituted Alkylhydroxy Derivatives of Cobalt Bis(1,2-Dicarbollide), Versatile Building Blocks for Synthetic Purposes. Dalton Trans..

[81] Nekvinda J., Šícha V., Hnyk D., Grüner B. (2014). Synthesis, Characterisation and Some Chemistry of C- and B-Substituted Carboxylic Acids of Cobalt Bis(Dicarbollide). Dalton Trans..

[82] Stogniy M.Y., Kazheva O.N., Chudak D.M., Shilov G.V., Filippov O.A., Sivaev I.B., Kravchenko A.V., Starodub V.A., Buravov L.I., Bregadze V.I. (2020). Synthesis and Study of *C* -Substituted Methylthio Derivatives of Cobalt Bis(Dicarbollide). RSC Adv..

[83] Hlatky G.G., Eckman R.R., Turner H.W. (1992). Metallacarboranes as Labile Anions for Ionic Zirconocene Olefin Polymerization Catalysts. Organometallics.

[84] DuBay W.J., Grieco P.A., Todd L.J. (1994). Lithium Cobalt-Bis-Dicarbollide: A Novel Lewis Acid Catalyst for the Conjugate Addition of Silyl Ketene Acetals to Hindered.alpha.,.beta.-Unsaturated Carbonyl Compounds. J. Org. Chem..

[85] Grieco P.A., DuBay W.J., Todd L.J. (1996). Lithium Cobalt-Bis-Dicarbollide Catalyzed Substitution Reactions of Allylic Acetates. Tetrahedron Letters.

[86] Nechai G.V., Sokolovskii F.S., Chuiko S.V. (2009). Catalytic Effect of Bis(Carbollyl) Complexes of Metals on the Combustion of Condensed Systems. Russ. J. Phys. Chem. B.

[87] Zhu Y., Chuanzhao L., Sudarmadji M., Hui Min N., Biying A.O., Maguire J.A., Hosmane N.S. (2012). An Efficient and Recyclable Catalytic System Comprising Nanopalladium(0) and a Pyridinium Salt of Iron Bis(Dicarbollide) for Oxidation of Substituted Benzyl Alcohol and Lignin. ChemistryOpen.

[88] Biying A.O., Yuanting K.T., Hosmane N.S., Zhu Y. (2017). Ionic Composite of Palladium(II)/Iron Bis(Dicarbollide) for Catalytic Oxidative Carbonylation in the Formation of Diphenyl Carbonate. J. Organomet. Chem..

[89] Nar I., Atsay A., Gümrükçü S., Karazehir T., Hamuryudan E. (2018). Low-Symmetry Phthalocyanine Cobalt Bis(Dicarbollide) Conjugate for Hydrogen Reduction. Eur. J. Inorg. Chem..

[90] Guerrero I., Kelemen Z., Viñas C., Romero I., Teixidor F. (2020). Metallacarboranes as Photoredox Catalysts in Water. Chem. A Eur. J..

[91] Guerrero I., Saha A., Xavier J.A.M., Viñas C., Romero I., Teixidor F. (2020). Noncovalently Linked Metallacarboranes on Functionalized Magnetic Nanoparticles as Highly Efficient, Robust, and Reusable Photocatalysts in Aqueous Medium. ACS Appl. Mater. Interfaces.

[92] Guerrero I., Viñas C., Romero I., Teixidor F. (2021). A Stand-Alone Cobalt Bis(Dicarbollide) Photoredox Catalyst Epoxidates Alkenes in Water at Extremely Low Catalyst Load. Green Chem..

[93] Gilman H., Cartledge F.K. (1964). The Analysis of Organolithium Compounds. J. Organomet. Chem..

[94] Heřmánek S. (1992). Boron-11 NMR Spectra of Boranes, Main-Group Heteroboranes, and Substituted Derivatives. Factors Influencing Chemical Shifts of Skeletal Atoms. Chem. Rev..

[95] Heřmánek S. (1999). NMR as a Tool for Elucidation of Structures and Estimation of Electron Distribution in Boranes and Their Derivatives. Inorganica Chim. Acta.

[96] Pfüller U., Franz H., Preiss A. (1977). Sudan Black B: Chemical Structure and Histochemistry of the Blue Main Components. Histochemistry.

[97] Fredricsson B., Laurent T.C., Lüning B. (1958). Decomposition of Sudan Black B Causing an Artefact in the Staining of Lipids. Stain. Technol..

[98] Lansink A.G.W. (1968). Thin Layer Chrornatography and Histochemistry of Sudan Black B. Histochemie.

[99] Grüner B., Plzák Z. (1997). High-Performance Liquid Chromatographic Separations of Boron-Cluster Compounds. J. Chromatogr. A.

[100] XcaliburTM. https://www.thermofisher.com/order/catalog/product/OPTON-30965?SID=srch-srp-OPTON-30965.

[101] (2018). APEX3, SAINT and SADABS.

[102] Palatinus L., Chapuis G. (2007). *SUPERFLIP*—A Computer Program for the Solution of Crystal Structures by Charge Flipping in Arbitrary Dimensions. J. Appl. Crystallogr..

[103] Betteridge P.W., Carruthers J.R., Cooper R.I., Prout K., Watkin D.J. (2003). *CRYSTALS* Version 12: Software for Guided Crystal Structure Analysis. J. Appl. Crystallogr..

[104] Rohlíček J., Hušák M. (2007). *MCE2005*—A New Version of a Program for Fast Interactive Visualization of Electron and Similar Density Maps Optimized for Small Molecules. J. Appl. Crystallogr..

[105] Delta NMR Software. https://www.jeol.com/products/scientific/nmr_software/Delta5.php.

[106] Eager SmartTM. https://www.thermofisher.com/order/catalog/product/11206100.

