# Supplementary material for: Halogenated Cobalt Bis-Dicarbollide Strong Acids as Reusable Homogeneous Catalysts for Fatty Acid Esterification with Methanol or Ethanol
Source: Int J Mol Sci. 2024 Dec 10;25(24):13263. doi: 10.3390/ijms252413263 (PMC11675812; doi:10.3390/ijms252413263)
Supplement: Supplementary file 1 [file ijms-25-13263-s001.zip › ijms-3325029-supplementary.pdf]

SUPPLEMENTARY INFORMATION

Halogenated Cobalt Bis-Dicarbollide Strong Acids as Reusable Homogeneous Catalysts for Fatty Acids Esterification with Methanol or Ethanol

Pavel Kaule [1], Václav Šícha [1], Jan Macháček [2], Yelizaveta Naumkina [3], Jan Čejka [3]

[1] *Jan Evangelista Purkyně University in Ústí nad Labem, Faculty of Science, Department of Chemistry, Pasteurova 3632/15, 40096 Ústí nad Labem, Czechia.*

[2] *Czech Academy of Sciences, Institute of Inorganic Chemistry, Department of Syntheses, Hlavní 1001, Řež, Czechia.*

[3] *Department of Solid State Chemistry, University of Chemistry and Technology, Prague, Technická 5, Prague, Czechia.*

** Correspondence author´s e-mail: jan.cejka@vscht.cz*

TABLE OF CONTENTS **Page**

Figure S1. Structural formula of [**1**$^-$] and its halogenderivatives. 3

Table S1. An overview of elemental CH combustion analyses results. 3

Table S2. An overview of obtained ESI MS data. 3

Figure S2. MS (ESI$^-$) zoomed experimental spectrum (top) of prepared Cs[Cl_4-**1**$^-$]. 4

Figure S3. MS (ESI$^-$) zoomed experimental spectrum (top) of prepared Cs[Br_4-**1**$^-$]. 4

Figure S4. HPLC TIC chromatogram of the Cs[Cl_4-**1**$^-$] accompanied by Cs[Cl_5-**1**$^-$]. 4

Figure S5. ESI MS spectra of the Cs[Cl_4-**1**$^-$]accompanied by Cs[Cl_5-**1**$^-$]. 5

Figure S6. UV-Vis spectra of the Cs[Cl_4-**1**$^-$]accompanied by Cs[Cl_5-**1**$^-$]. 5

Figure S7. HPLC TIC chromatogram of the Cs[Br_4-**1**$^-$] accompanied by Cs[Br_5-**1**$^-$]. 6

Figure S8. ESI MS spectra of the Cs[Br_4-**1**$^-$] accompanied by Cs[Br_5-**1**$^-$]. 6

Figure S9. UV-Vis spectra of the Cs[Br_4-**1**$^-$] accompanied by Cs[Br_5-**1**$^-$]. 7

Figure S10. The HPLC PDA chromatogram of the comparative mixture of all studied catalysts. 7

Figure S11. ESI MS spectra of the comparative mixture of all studied catalysts. 8

Figure S12. UV-Vis spectra of the comparative mixture of all studied catalysts. 8

Figure S13. Overlapped UV-Vis spectra of all used acids without an indicator. 9

Figure S14. Overlapped UV-Vis spectra of all used acids after reaction with the SUDAN Black B. 9

Figure S15. Overlapped UV-Vis spectra of H_2SO_4 and all heteroborane acids. 9

Figure S16. HPLC APCI MS TIC chromatograms of the esterification with 1 mol% of H_3O[Cl_2-**1**$^-$]. 10

Figure S17. Acids conversions with methanol and 3 mol.% of the catalyst under desiccation. 11

Figure S18. Acids conversions with ethanol and 3 mol.% of the catalyst under desiccation. 11

Figure S19. Acids conversions with methanol and 2 mol.% of the catalyst under desiccation. 11

Figure S20. Acids conversions with ethanol and 2 mol.% of the catalyst under desiccation. 11

Table S3. TON/TOF benchmarks of H_3O[Cl_2-**1**$^-$] after esterification with methanol. 11

Table S4. TON/TOF benchmarks of $H_3O[HSO_4]$ after esterification with methanol. 11

Table S5. TON/TOF benchmarks of $H_3O[Cl_2$-**1**$^-]$ after esterification with ethanol. 11

Table S6. TON/TOF benchmarks of $H_3O[HSO_4]$ after esterification with ethanol. 11

Table S7. TON activity benchmark of the triple used $H_3O[Cl_2$-**1**$^-]$. 12

Table S8. TOF activity benchmark of the triple used $H_3O[Cl_2$-**1**$^-]$. 12

Figure S21. $^{1}H\{^{11}B\}$ NMR spectrum of Cs[Cl_4-**1**$^-$]. 12

Figure S22. $^{13}C\{^{1}H\}$ NMR spectrum of Cs[Cl_4-**1**$^-$]. 13

Figure S23. $^{11}B\{^{1}H\}$ (top) and ^{11}B (down) NMR spectrum of Cs[Cl_4-**1**$^-$]. 13

Figure S24. ^{11}B -$^{11}B\{^{1}H\}$ COSY NMR spectrum of Cs[Cl_4-**1**$^-$]. 14

Figure S25. ^{1}H-^{11}B HMQC NMR spectrum of Cs[Cl_4-**1**$^-$]. 14

Figure S26. $^{1}H\{^{11}B\}$ NMR spectrum of Cs[Br_4-**1**$^-$]. 15

Figure S27. $^{13}C\{^{1}H\}$ NMR spectrum of Cs[Br_4-**1**$^-$]. 15

Figure S28. ^{11}B (down) and $^{11}B\{^{1}H\}$ (top) NMR spectrum of Cs[Br_4-**1**$^-$]. 16

Figure S29. $^{11}B\{^{1}H\}$ -$^{11}B\{^{1}H\}$ COSY NMR spectrum of Cs[Br_4-**1**$^-$]. 16

Figure S30. ^{1}H-^{11}B HMQC NMR spectrum of Cs[Br_4-**1**$^-$]. 17

Figure S31. ^{23}Na NMR signal increase of Na^+ extracted from the 3Å zeolite by hot methanol. 17

Figure S32. ^{23}Na NMR experiment of methanol refluxed under argon inert without zeolite. 18

Figure S33. ^{23}Na NMR signal increase as a proof of Na^+ extracted from the 3Å zeolite by hot ethanol. 18

Figure S34. ^{23}Na NMR experiment of ethanol refluxed under argon inert without zeolite. 19

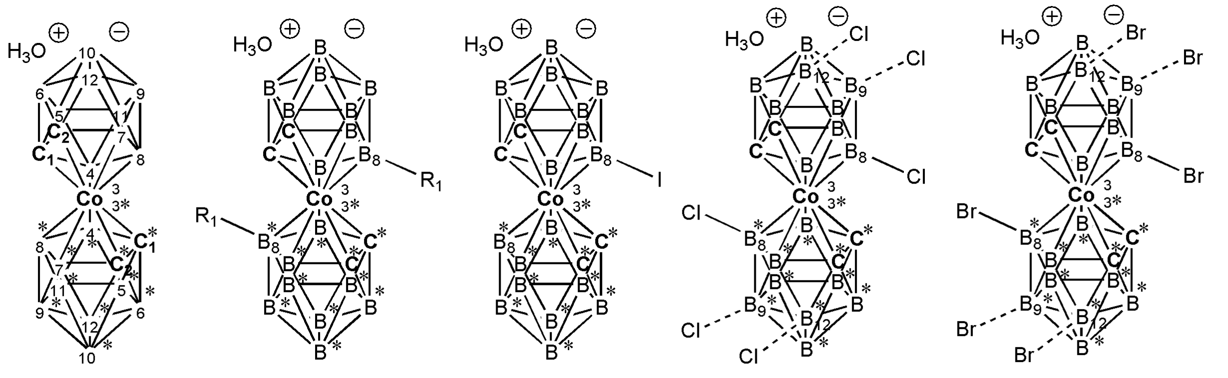

[1⁻] [F_2-1⁻], [Cl_2-1⁻], [Br_2-1⁻] [I-1⁻] [Cl_4-1⁻] [Br_4-1⁻]

Figure S1. Structural formula of [**1**⁻] and its halogenderivatives in the series studied. R_1 represents F or Cl or Br. Cage terminal H atoms attached to B and C vertices were omitted for clarity.

Sample	formula	C cal. [%]	C exp. [%]	SD [%]	C exp.-calc. [%]	H cal. [%]	H exp. [%]	SD [%]	H exp.-cal. [%]
Cs[**1**⁻]	$B_{18}C_4H_{22}CoCs$	10.52	10.41	0.12	-0.11	4.86	4.84	0.21	-0.02
K[F_2-**1**⁻]	$B_{18}C_4H_{20}F_2CoK$	12.05	12.34	0.25	0.29	5.05	5.31	0.23	0.26
Cs[Cl_2-**1**⁻]	$B_{18}C_4H_{20}Cl_2CoCs$	9.14	9.23	0.65	0.09	3.84	3.78	0.33	-0.06
K[Br_2-**1**⁻]	$B_{18}C_4H_{20}Br_2CoK$	9.23	9.53	0.03	0.30	3.87	3.72	0.15	-0.15
Cs[Cl_4-**1**⁻]	$B_{18}C_4H_{18}Cl_4CoCs$	8.08	8.74	0.15	0.66	3.05	3.33	0.20	0.28
Cs[Br_4-**1**⁻]	$B_{18}C_4H_{18}Br_4CoCs$	6.22	7.70	0.98	1.48	2.35	2.46	0.04	0.11
Cs[I_1-**1**⁻] C_6H_6 solvate	$B_{18}C_4H_{21}ICoCs$ + 0.5 C_6H_6	13.53	13.85	0.07	0.32	3.89	4.25	0.09	0.36

Table S1. An overview of elemental CH combustion analyses results of prepared hydrophobic salts obtained with Flash*Smart* analyzer (Thermo Scientific Inc., USA).

**Anion [M]⁻**	**Formula**	***m/z* calc.** 100%	***m/z* exp.** 100%	**MS² CID** eV
[**1**⁻]	$B_{18}C_4Co_1H_{22}$	324.28	324.30	95
[F_2-**1**⁻]	$B_{18}C_4Co_1F_2H_{20}$	360.26	360.28	100
[F_3-**1**⁻]	$B_{18}C_4Co_1F_3H_{19}$	378.25	378.26	101
[Cl_2-**1**⁻]	$B_{18}C_4Cl_2Co_1H_{20}$	392.21	392.25	114
[Br_2-**1**⁻]	$B_{18}Br_2C_4Co_1H_{20}$	480.09	480.10	70
[I_1-**1**⁻]	$B_{18}C_4Co_1H_{21}I_1$	450.18	450.20	105
[I_2-**1**⁻]	$B_{18}C_4Co_1H_{20}I_2$	576.08	576.08	94
[Cl_4-**1**⁻]	$B_{18}C_4Cl_4Co_1H_{17}$	461.12	461.16	107
[Cl_5-**1**⁻]	$B_{18}C_4Cl_5Co_1H_{17}$	496.09	496.10	107
[Br_4-**1**⁻]	$B_{18}Br_4C_4Co_1H_{18}$	639.92	639.92	102
[Br_5-**1**⁻]	$B_{18}Br_5C_4Co_1H_{17}$	718.84	718.82	102

Table S2. An overview of obtained ESI MS data. MS² CID represents energy quantum used for the succesful fragmentation of the respective molecular ion during the collision induced dissociation (CID) experiment.

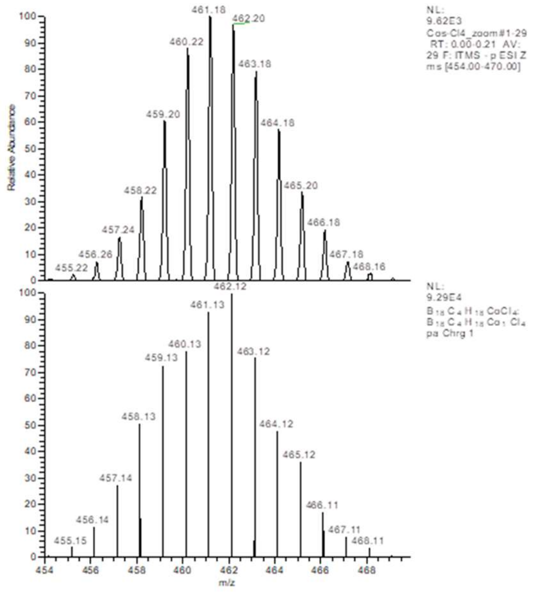

Figure S2. MS (ESI-) zoomed experimental spectrum (top) of prepared mixture of isobaric monoanions Cs[8,8′,9,9′-Cl_4-**1**-] and Cs[8,8′,12,12′-Cl_4-**1**-] measured in acetonitrile in comparison with the calculated spectrum (down).

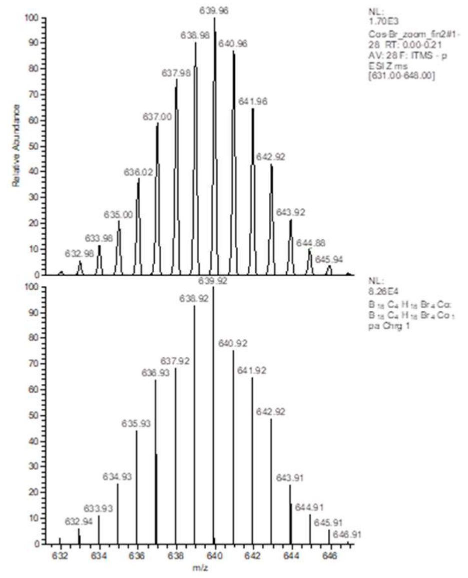

Figure S3. MS (ESI-) zoomed experimental spectrum (top) of prepared mixture of isobaric monoanions Cs[8,8′,9,9′-Br_4-**1**-] and Cs[8,8′,12,12′-Br_4-**1**-] measured in acetonitrile in comparison with the calculated spectrum (down).

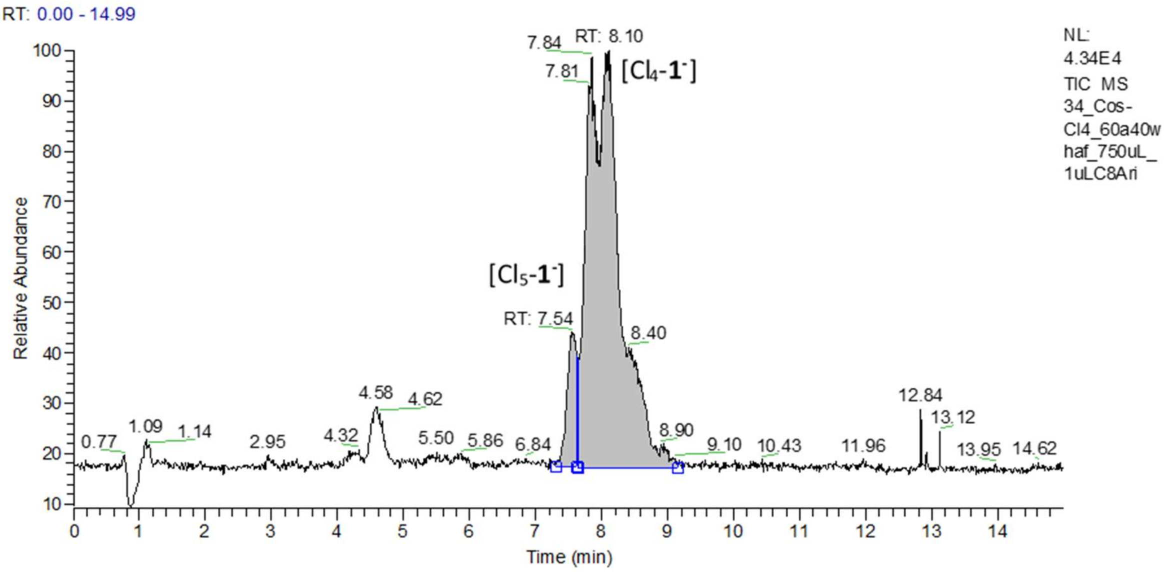

Figure S4. HPLC TIC chromatogram of the Cs[8,8′,9,9′-Cl_4-**1**-] in the mixture with equal ammount of Cs[8,8′,12,12′-Cl_4-**1**-], accompanied by minor Cs[Cl_5-**1**-].

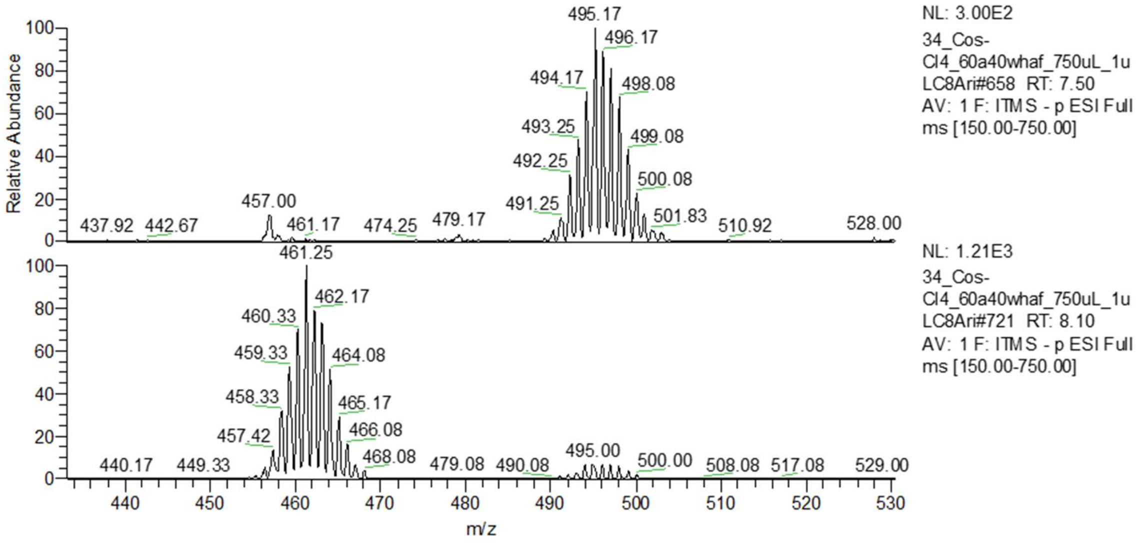

Figure S5. ESI MS spectra of the Cs[8,8′,9,9′-Cl_4-**1**$^-$] (7.50 min) in the mixture with equal ammount of Cs[8,8′,12,12′-Cl_4-**1**$^-$], accompanied by minor Cs[Cl_5-**1**$^-$] (8.10 min).

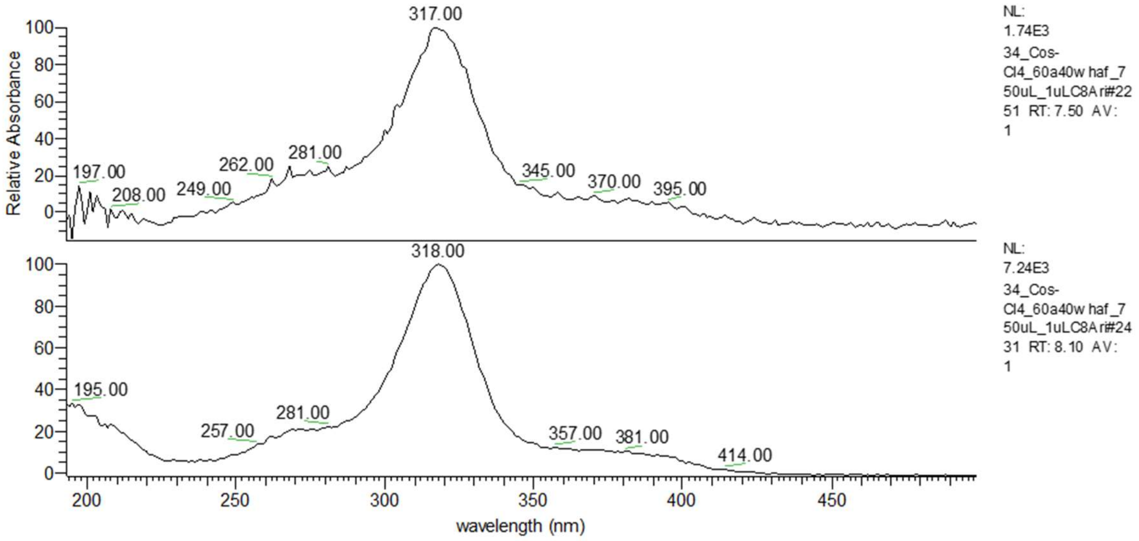

Figure S6. UV-Vis spectra of the Cs[8,8′,9,9′-Cl_4-**1**$^-$] (7.50 min) in the mixture with equal ammount of Cs[8,8′,12,12′-Cl_4-**1**$^-$], accompanied by minor Cs[Cl_5-**1**$^-$] (8.10 min).

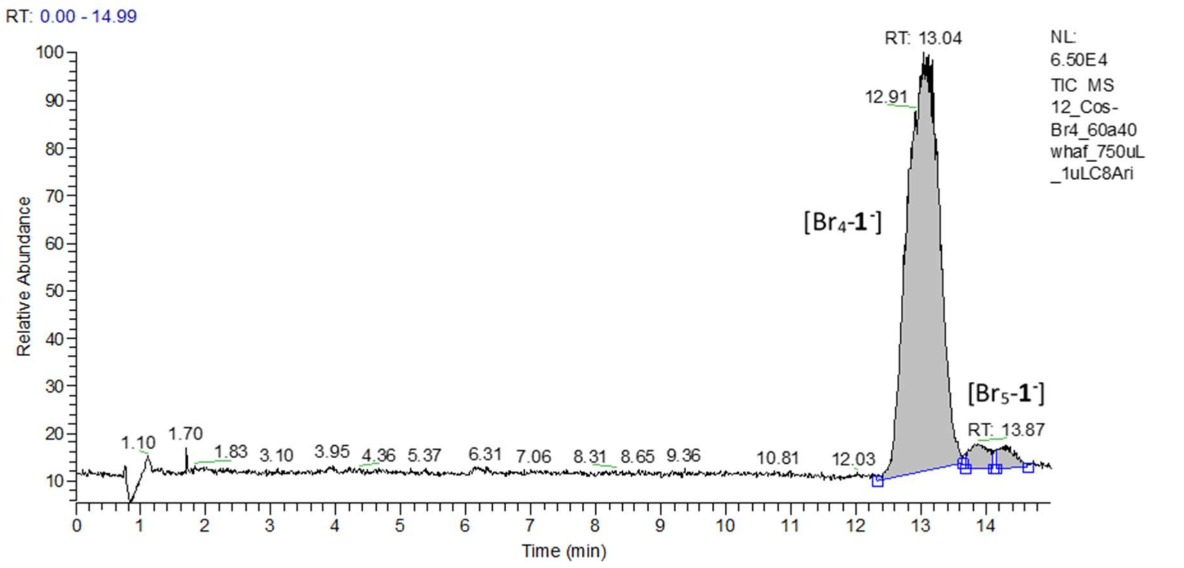

Figure S7. HPLC TIC chromatogram of the Cs[8,8′,9,9′-Br$_4$-**1**$^-$]$^-$ in the mixture with equal ammount of Cs[8,8′,12,12′-Br$_4$-**1**$^-$], accompanied by minor Cs[Br$_5$-**1**$^-$].

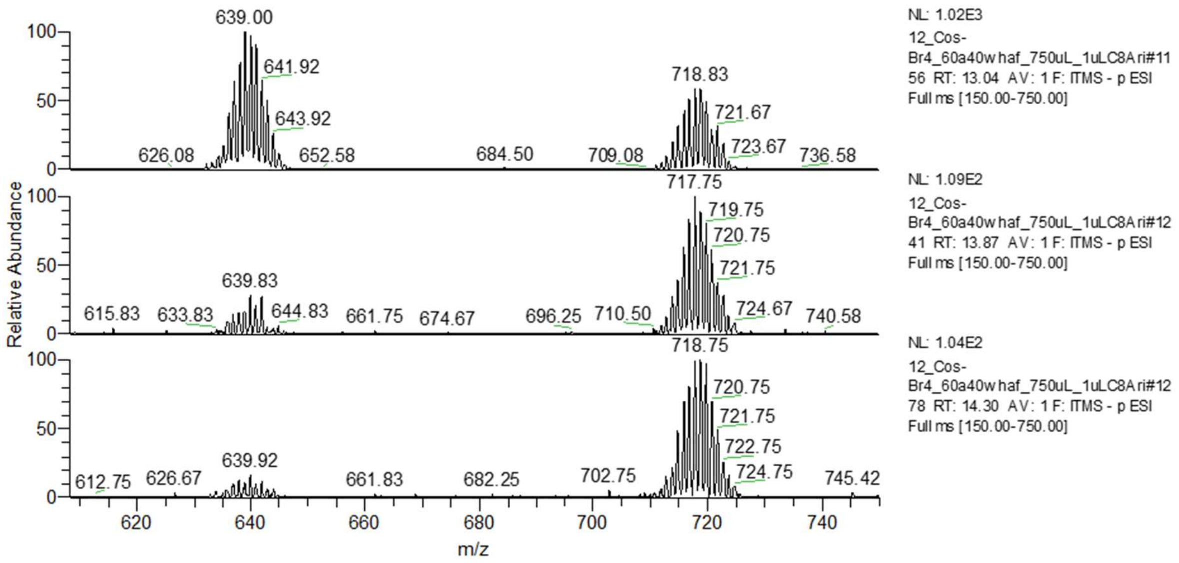

Figure S8. ESI MS spectra of the Cs[8,8′,9,9′-Br$_4$-**1**$^-$]$^-$ in the mixture with equal ammount of Cs[8,8′,12,12′-Br$_4$-**1**$^-$], accompanied by minor Cs[Br$_5$-**1**$^-$] measured at 13.04, 13.87 and 14.30 min.

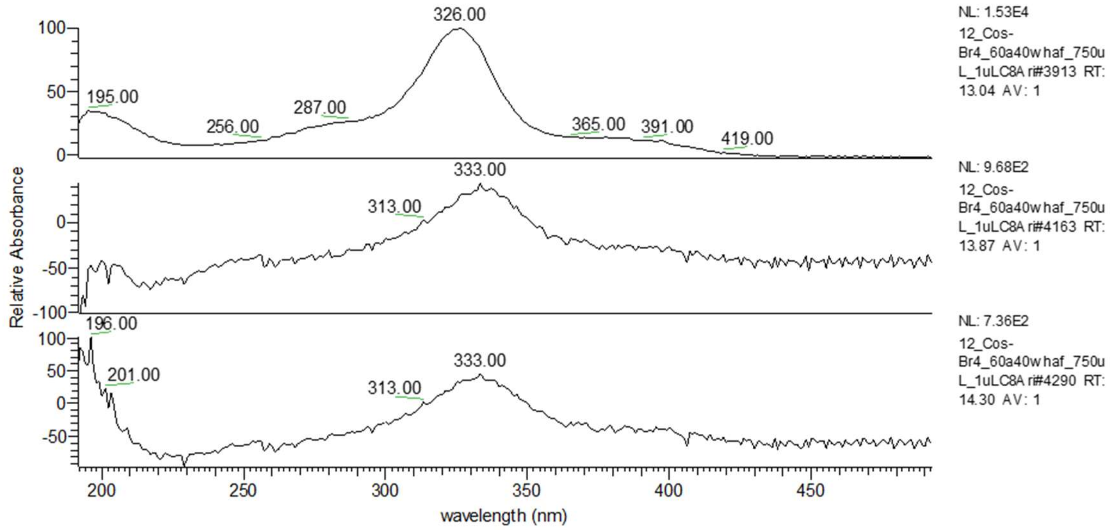

Figure S9. UV-Vis spectra of the Cs[8,8′,9,9′-Br_4-**1**$^-$]$^-$ in the mixture with equal ammount of Cs[8,8′,12,12′-Br_4-**1**$^-$], accompanied by minor Cs[Br_5-**1**$^-$] measured at 13.04, 13.87 and 14.30 min.

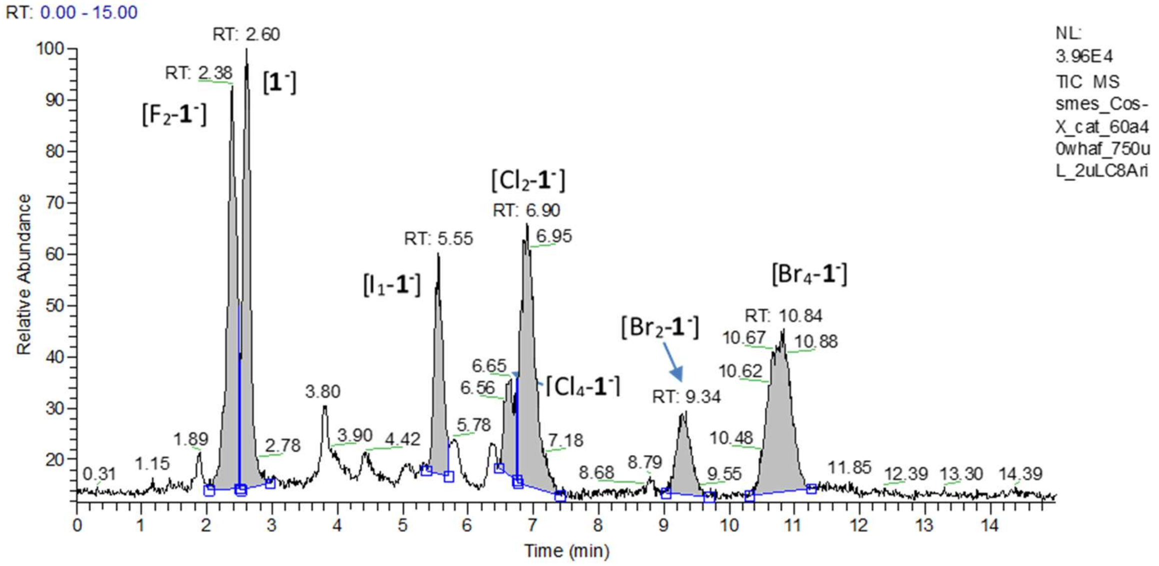

Figure S10. The HPLC PDA chromatogram of the comparative mixture of all studied catalysts.

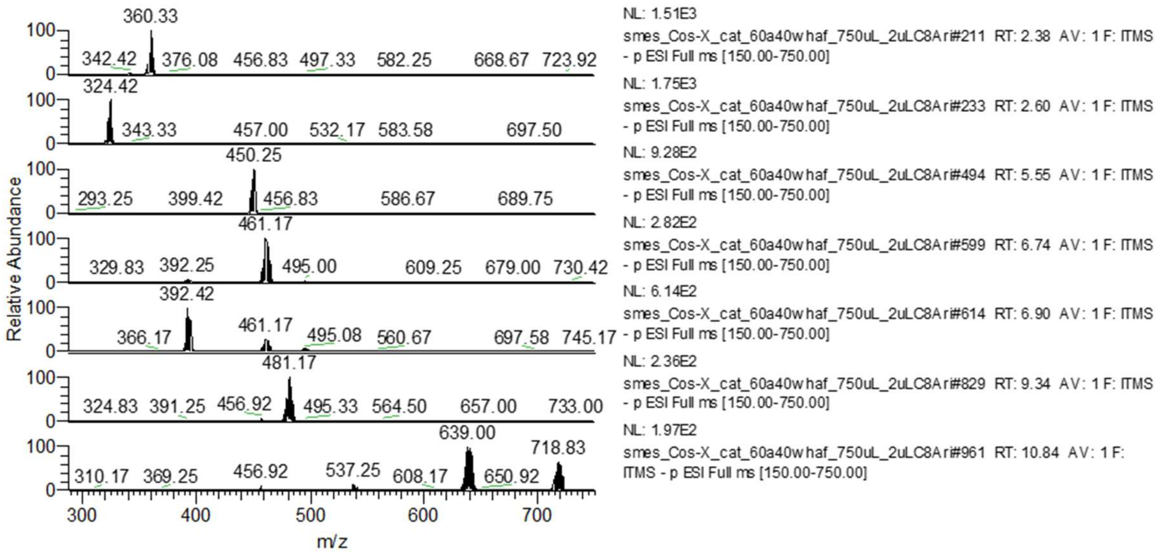

Figure S11. ESI MS spectra of the comparative mixture of all studied catalysts.

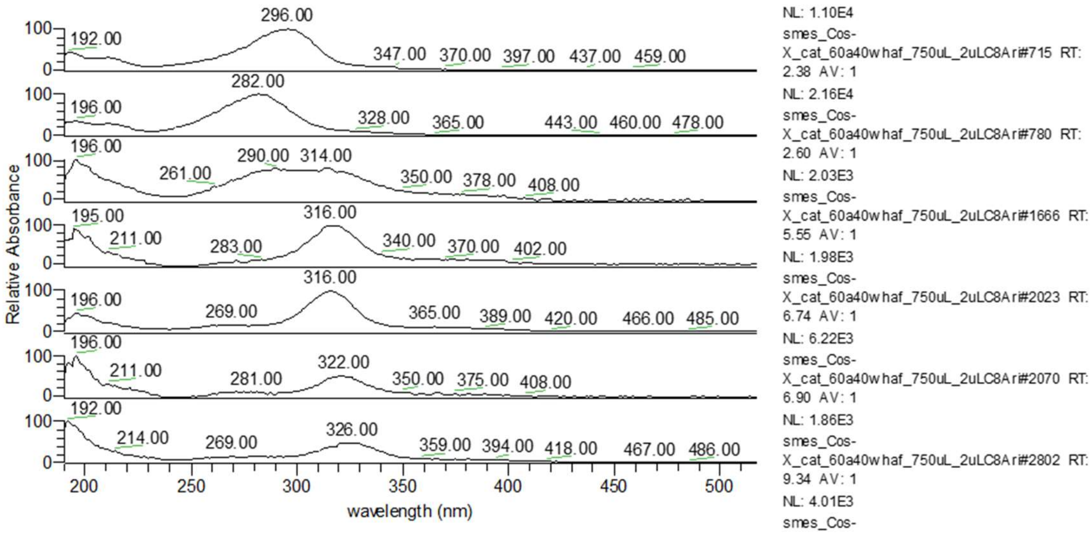

Figure S12. UV-Vis spectra of the comparative mixture of all studied catalysts.

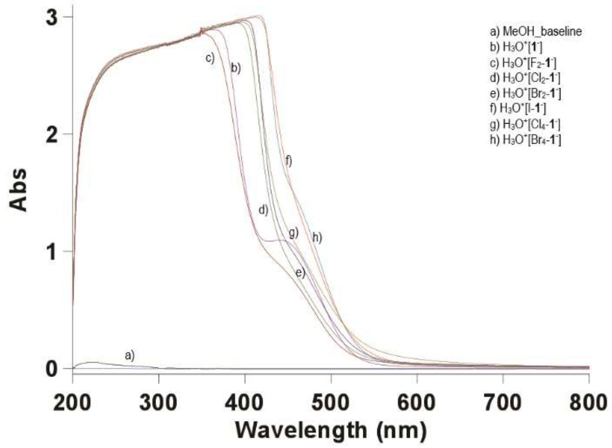

Figure S13. Overlapped UV-Vis spectra of all used acids (5 mmol) without an indicator measured in pure methanol (uHPLC grade, 0.98±0.13 mg H_2O/g of solvent) at 25°C.

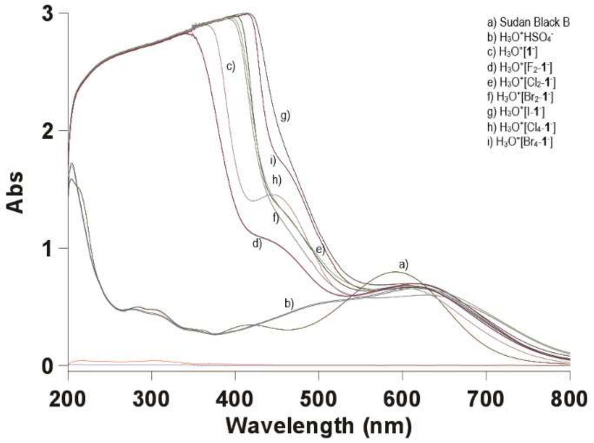

Figure S14. Overlapped UV-Vis spectra of all used acids ($5x10^{-3}$ mol) after reaction with the SUDAN Black B indicator (UV_{max} = 592 nm; $7.2x10^{-5}$ mol) measured in the mixture of methanol/water (75/25, v/v) at 25°C.

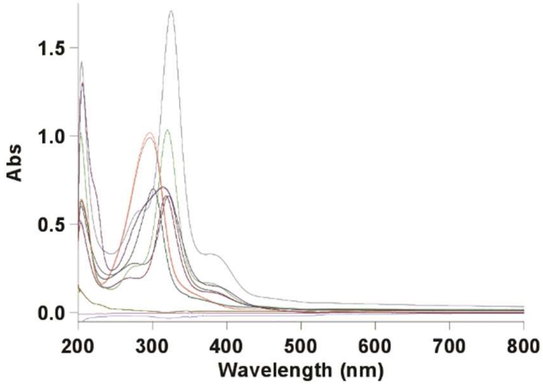

Figure S15. Overlapped UV-Vis spectra of H_2SO_4 and all heteroborane acids based on [$\mathbf{1}^-$] (1 x 10^{-3} g.cm^{-3}) measured in pure methanol at 25°C.

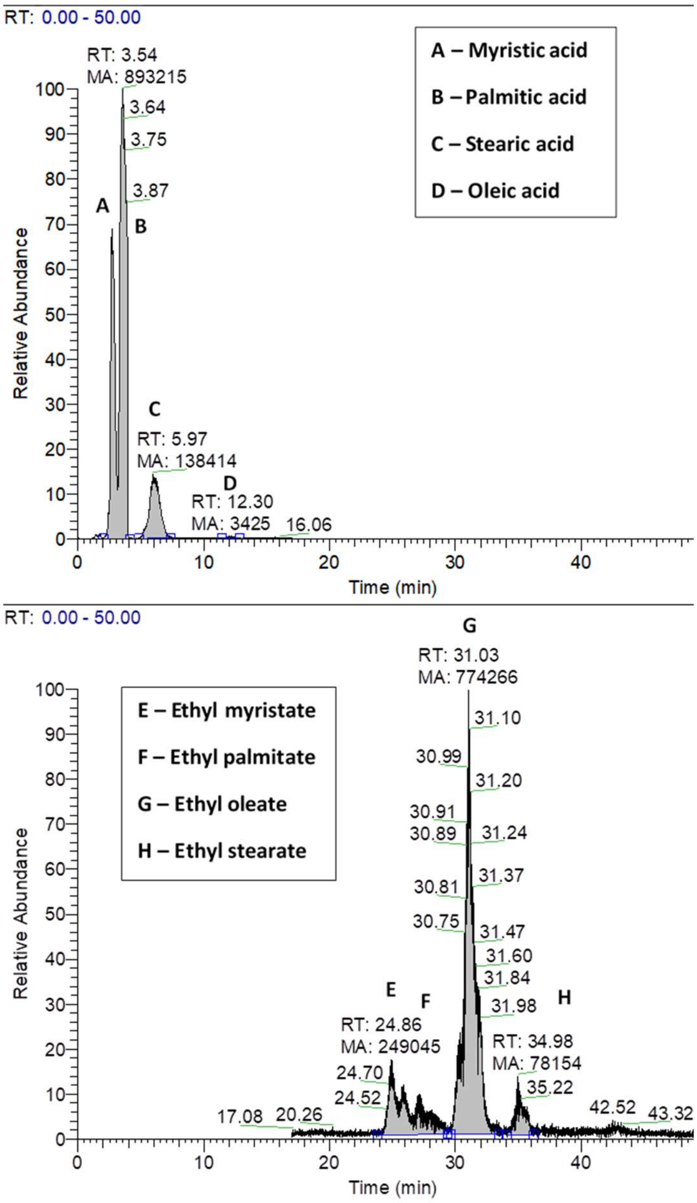

Figure S16. HPLC APCI MS TIC chromatograms of an esterification mixture of four fatty acids catalyzed by 1 mol% of $H_3O[Cl_2$-**1**$^-]$, after 2 h of reflux in continually desiccated ethanol. TIC chromatogram on the top represents isolated free fatty acids (anions) and the down chromatogram show separated esters (cations) making FAEE product.

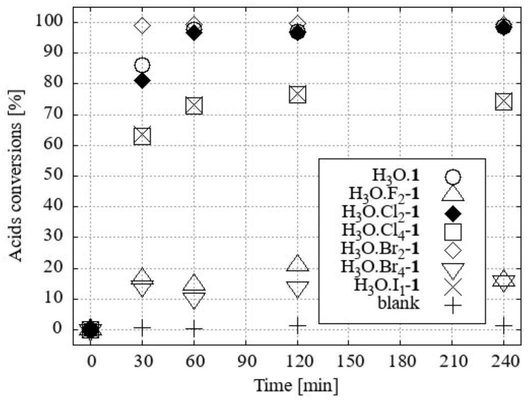

Figure S17. Acids conversions of FAME with methanol and 3 mol.% of the catalyst under desiccation.

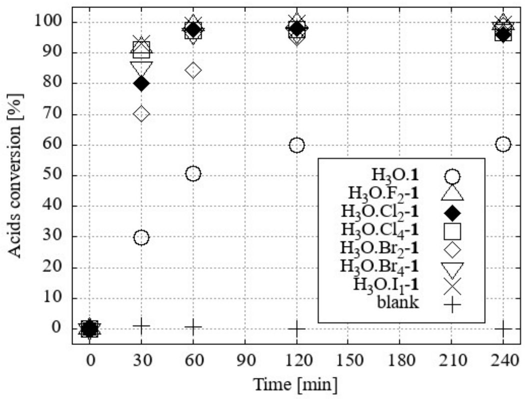

Figure S18. Acids conversions of FAEE with ethanol and 3 mol.% of the catalyst under desiccation.

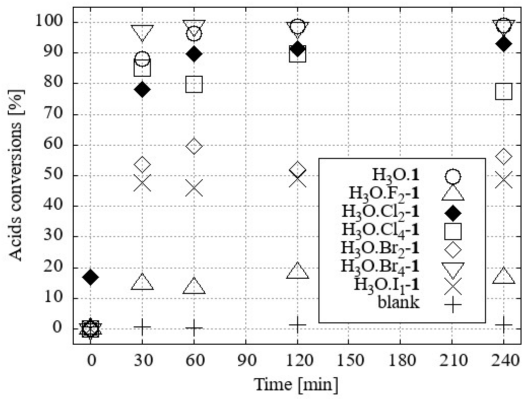

Figure S19. Acids conversions of FAME with methanol and 2 mol.% of the catalyst under desiccation.

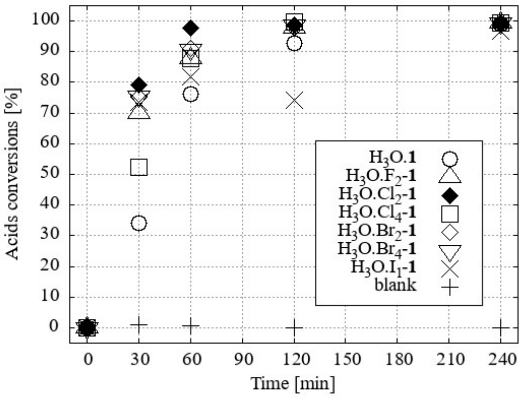

Figure S20. Acids conversions of FAEE with ethanol and 2 mol.% of the catalyst under desiccation.

	Time [min]	Conversion [%]	TON	TOF [s-1]
$H_3O[Cl_2$-**1**$^-]$	30	81,3	81,3	4,5E-02
MeOH	60	96,7	96,7	2,7E-02
	120	96,8	96,8	1,3E-02
	240	98,4	98,4	6,8E-03

Table S3. TON/TOF benchmarks of $H_3O[Cl_2$-**1**$^-]$ after esterification with methanol.

	Time [min]	Conversion [%]	TON	TOF $[s^{-1}]$
$H_3O[Cl_2$-**1**$^-]$	30	63,5	63,5	3,5E-02
EtOH	60	86,8	86,8	2,4E-02
	120	99,4	99,4	1,4E-02
	240	99,5	99,5	6,9E-03

Table S5. TON/TOF benchmarks of $H_3O[Cl_2$-**1**$^-]$ after esterification with ethanol.

	Time [min]	Conversion [%]	TON	TOF $[s^{-1}]$
$H_3O[HSO_4]$	30	80,3	80,3	4,5E-02
MeOH	60	88,5	88,5	2,5E-02
	120	98,1	98,1	1,4E-02
	240	99,3	99,3	6,9E-03

Table S4. TON/TOF benchmarks of $H_3O[HSO_4]$ after esterification with methanol.

	Time [min]	Conversion [%]	TON	TOF $[s^{-1}]$
$H_3O[HSO_4]$	30	29,8	29,8	1,7E-02
EtOH	60	85,6	85,6	2,4E-02
	120	98,9	98,9	1,4E-02
	240	99,6	99,6	6,9E-03

Table S6. TON/TOF benchmarks of $H_3O[HSO_4]$ after esterification with ethanol.

TON after 240 min		
catalyst $H_3O[Cl_2$-**1**$^-]$	MeOH	EtOH
1st reaction	98,4	99,5
2nd reaction	93,0	98,5
3rd reaction	84,9	99,2
**Sum**	**276,3**	**297,2**

Table S7. TON activity benchmark of the triple used $H_3O[Cl_2$-**1**$^-]$.

TOF after 240 min [s^{-1}]		
catatyst $H_3O[Cl_2$-**1**$^-]$	MeOH	EtOH
1st reaction	6,84E-03	6,91E-03
2nd reaction	6,46E-03	6,84E-03
3rd reaction	5,90E-03	6,89E-03

Table S8. TOF activity benchmark of the triple used $H_3O[Cl_2$-**1**$^-]$.

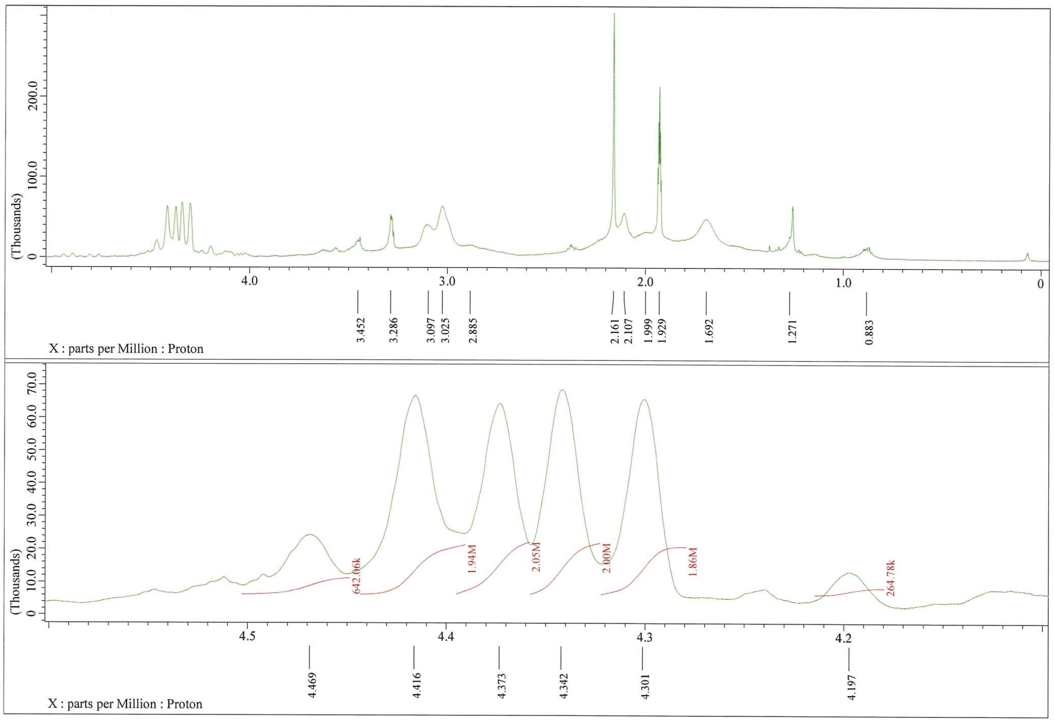

Figure S21. $^1H\{^{11}B\}$ spectrum of Cs[Cl_4-**1**$^-$] – mixture of dominant isomers Cs[8,8´,9,9´-Cl_4-**1**$^-$] and Cs[8,8´,12,12´-Cl_4-**1**$^-$].

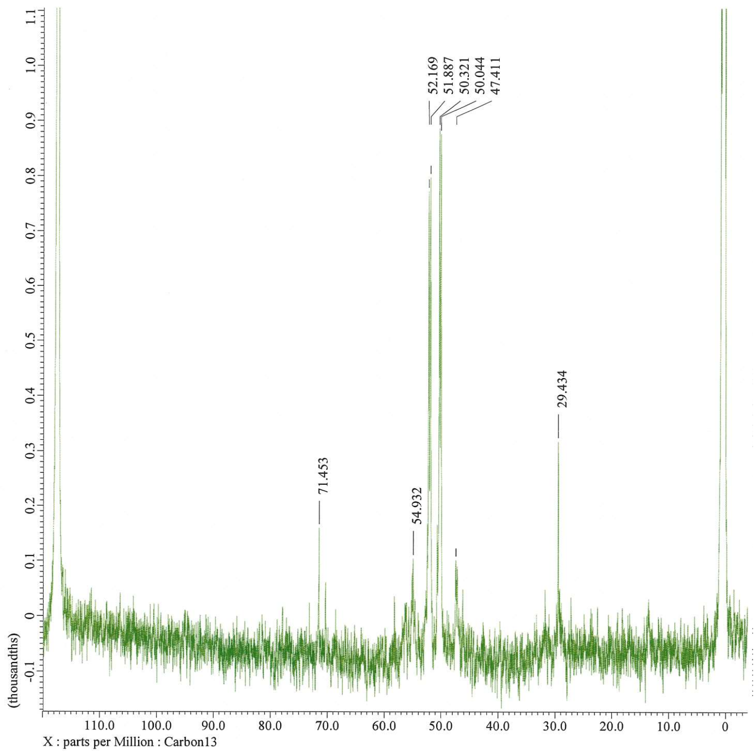

Figure S22. $^{13}C\{^{1}H\}$ NMR spectrum of Cs[Cl_4-**1**$^-$] – mixture of considered isomers Cs[8,8′,9,9′-Cl_4-**1**$^-$] and Cs[8,8′,12,12′-Cl_4-**1**$^-$] in CD_3CN at RT are represented by a splitted broaden singlets at 52.17 and 50.04 ppm.

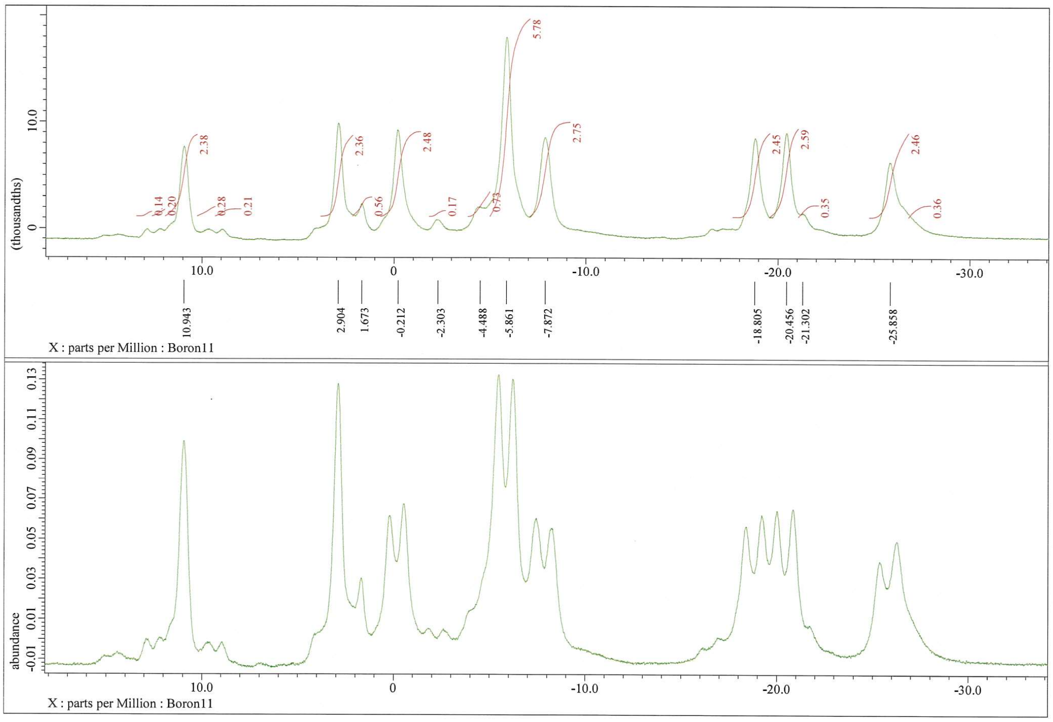

Figure S23. $^{11}B\{^{1}H\}$ (top) and ^{11}B (down) NMR spectrum of Cs[Cl_4-**1**$^-$] – mixture of Cs[8,8′,9,9′-Cl_4-**1**$^-$] and Cs[8,8′,12,12′-Cl_4-**1**$^-$] in CD_3CN at RT confirms that a moiety ratio of these isomers is equal. Small signals on the ^{11}B NMR spectrum baseline were tentatively identified as a less abundant equal mixture of Cs[7,8,8′,9,9′-Cl_5-**1**$^-$] and Cs[7,8,8′,12,12′-Cl_5-**1**$^-$].

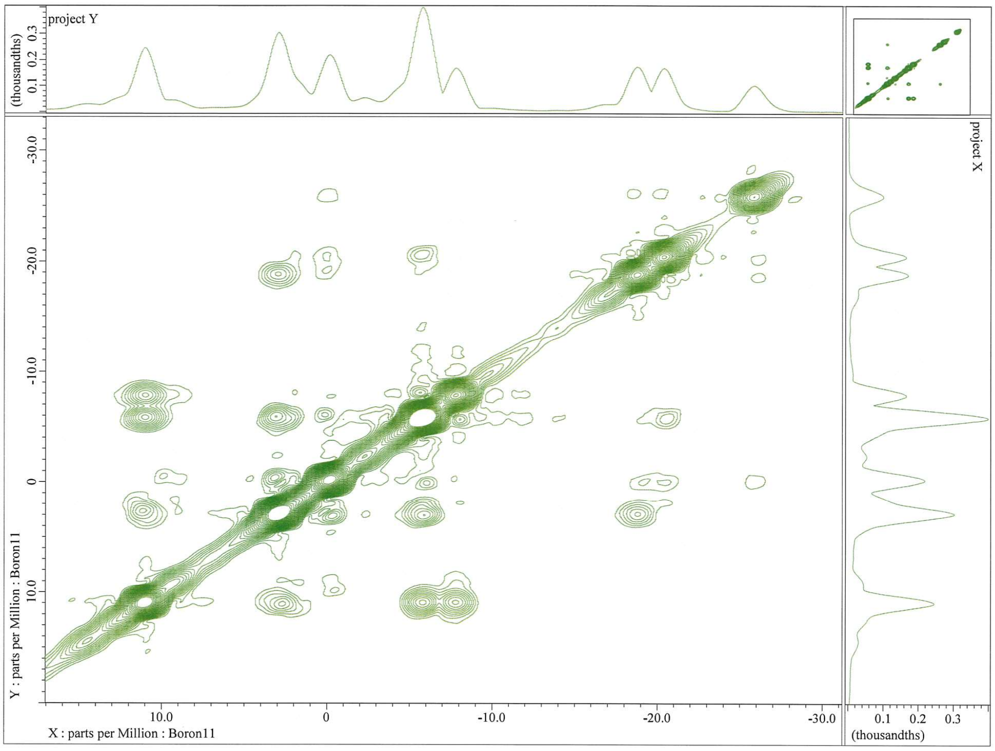

Figure S24. ^{11}B -$^{11}B\{^{1}H\}$ COSY NMR spectrum of Cs[Cl_4-**1**$^-$] – mixture of Cs[8,8´,9,9´-Cl_4-**1**$^-$] and Cs[8,8´,12,12´-Cl_4-**1**$^-$] in CD_3CN at RT. The experiment confirms that chlorine atoms substitute hydrogens on boron atoms 8,8´,9,9´ or 8,8´,12,12´.

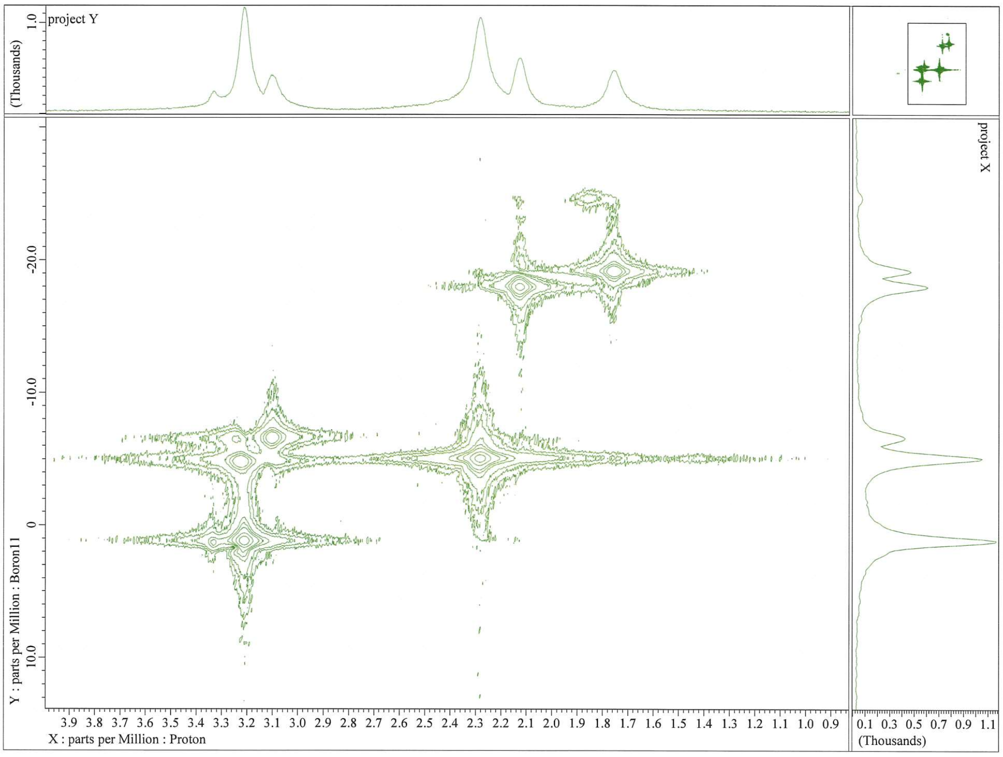

Figure S25. ^{1}H-^{11}B HMQC NMR spectrum of Cs[Cl_4-**1**$^-$] – Structure assignment of mixture of Cs[8,8´,9,9´-Cl_4-**1**$^-$] and Cs[8,8´,12,12´-Cl_4-**1**$^-$] is based on the 2D experiment assigned specific cluster hydroges attached to borons.

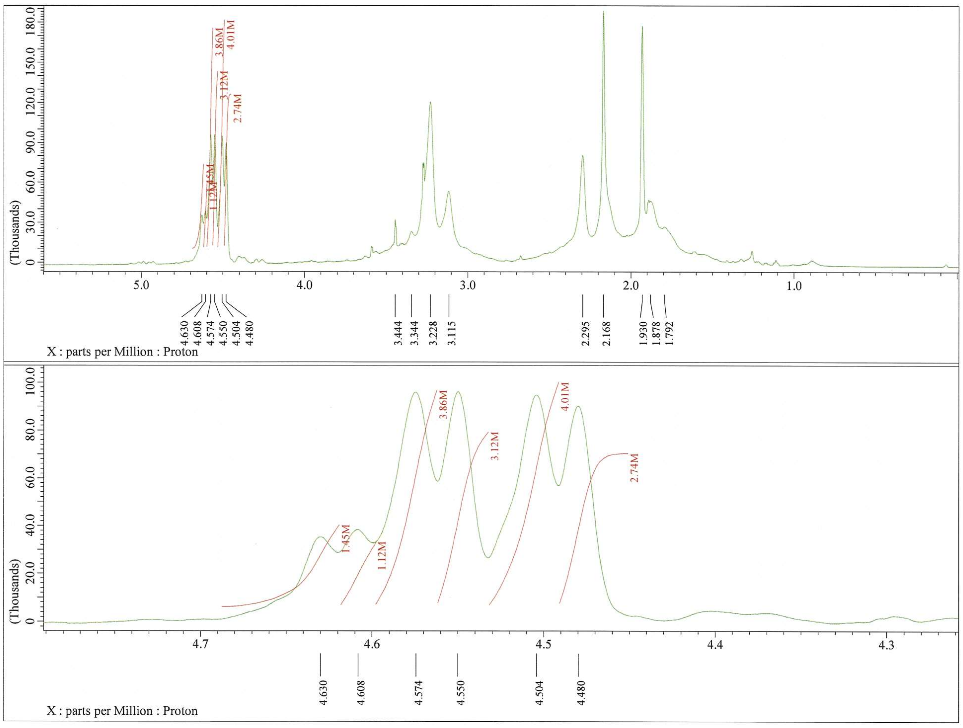

Figure S26. ^{1}H{^{11}B} NMR spectrum of Cs[Br$_4$-**1**$^-$] – mixture of Cs[8,8′,9,9′-Br$_4$-**1**$^-$] and Cs[8,8′,12,12′-Br$_4$-**1**$^-$] confirms that a moiety ratio of isomers is equal. The third pair of small intensity broaden singlets (4.63 and 4.61 ppm) suggests existence of the bromine atom attached to B7 in the pentagon opposition to skeletal CH carbon.

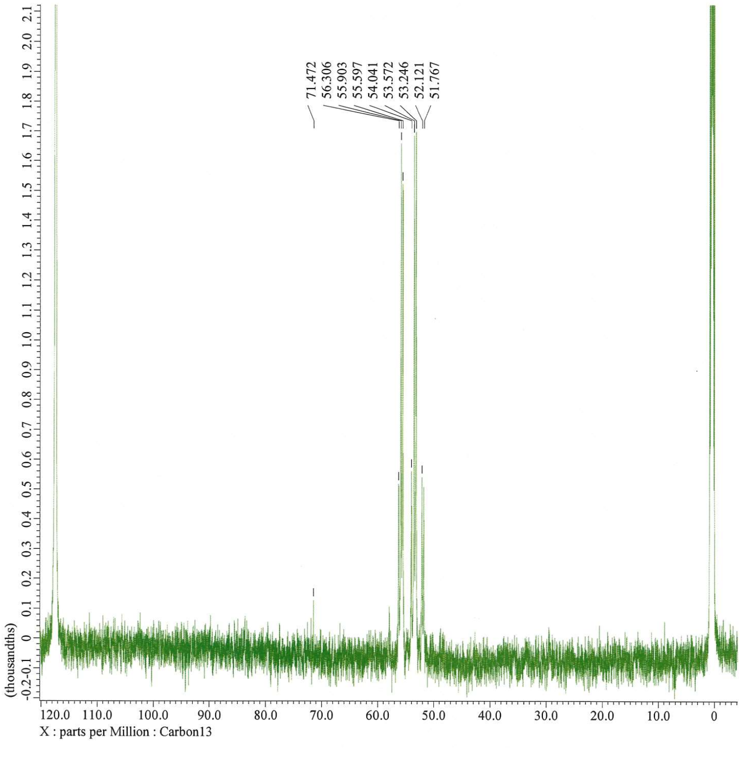

Figure S27. ^{13}C{^{1}H} NMR spectrum of Cs[Br$_4$-**1**$^-$] – mixture of Cs[8,8′,9,9′-Br$_4$-**1**$^-$] and Cs[8,8′,12,12′-Br$_4$-**1**$^-$]. Structures of considered positional isomers B8,8',9,9,' and B8,8',12,12' are represented by two broaden singlets at 55.90 or 54.04 ppm. Singlet 52.12 of two C-Br groups probably belongs to skeletal carbon in opposition to the bromine substited at B7 atom.

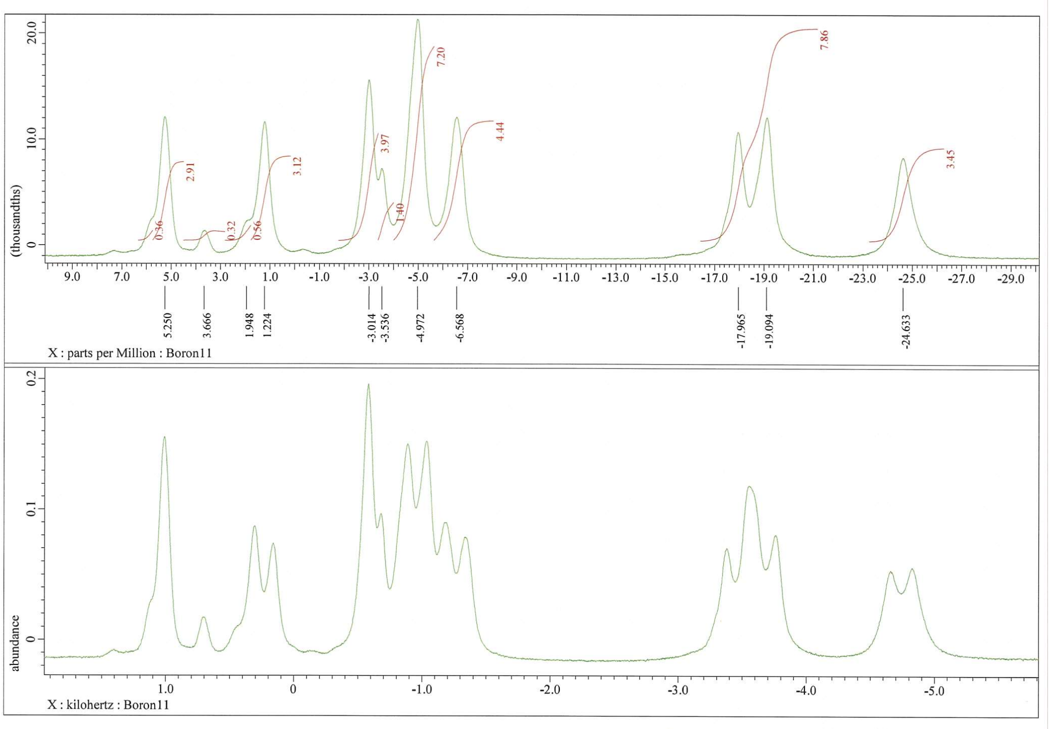

Figure S28. ^{11}B (down) and ^{11}B{^{1}H} (top) NMR spectrum of Cs[Br$_4$-**1**$^-$] – mixture of Cs[8,8′,9,9′-Br$_4$-**1**$^-$] and Cs[8,8′,12,12′-Br$_4$-**1**$^-$] in CD$_3$CN at RT confirms that a moiety ratio of these isomers is equal. Small signals on the ^{11}B NMR spectrum baseline were tentatively identified as an minor abundant equal mixture of Cs[7,8,8′,9,9′-Br$_5$-**1**$^-$] and Cs[7,8,8′,12,12′-Br$_5$-**1**$^-$].

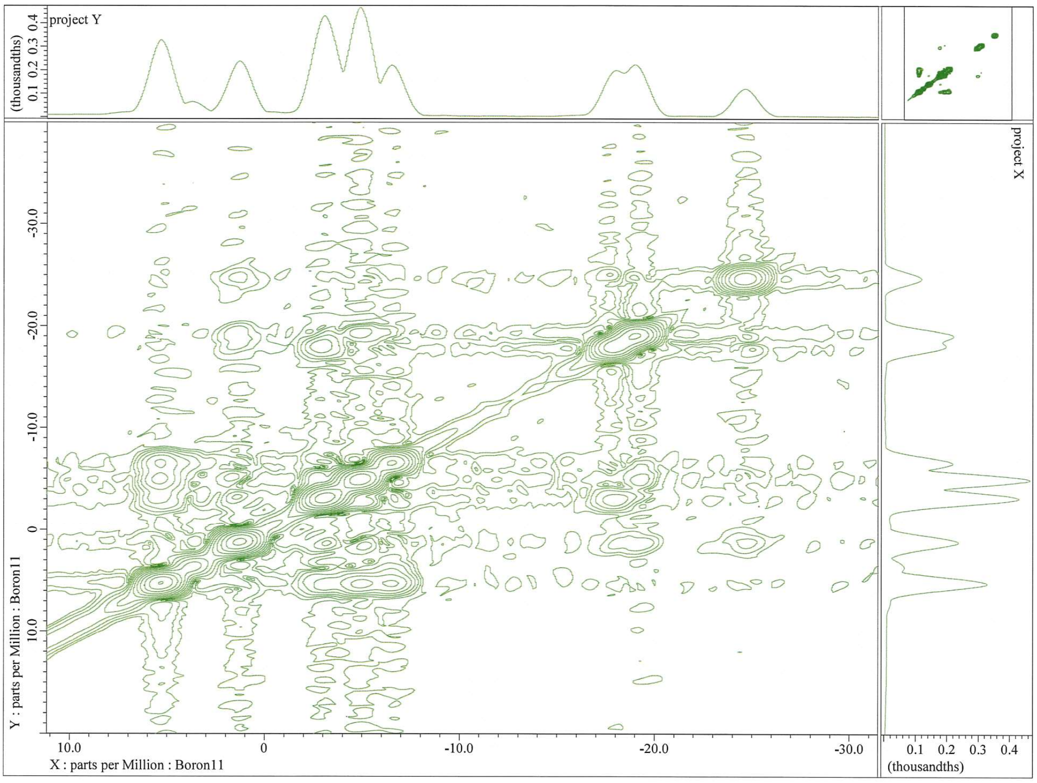

Figure S29. ^{11}B{^{1}H} -^{11}B{^{1}H} COSY NMR spectrum of Cs[Br$_4$-**1**$^-$] – mixture of Cs[8,8′,9,9′-Br$_4$-**1**$^-$] and Cs[8,8′,12,12′-Br$_4$-**1**$^-$] in CD$_3$CN at RT. Thanks this experiment was verified that bromine atoms too occupy the B vertex B8,8′and B9,9′or B12,12.

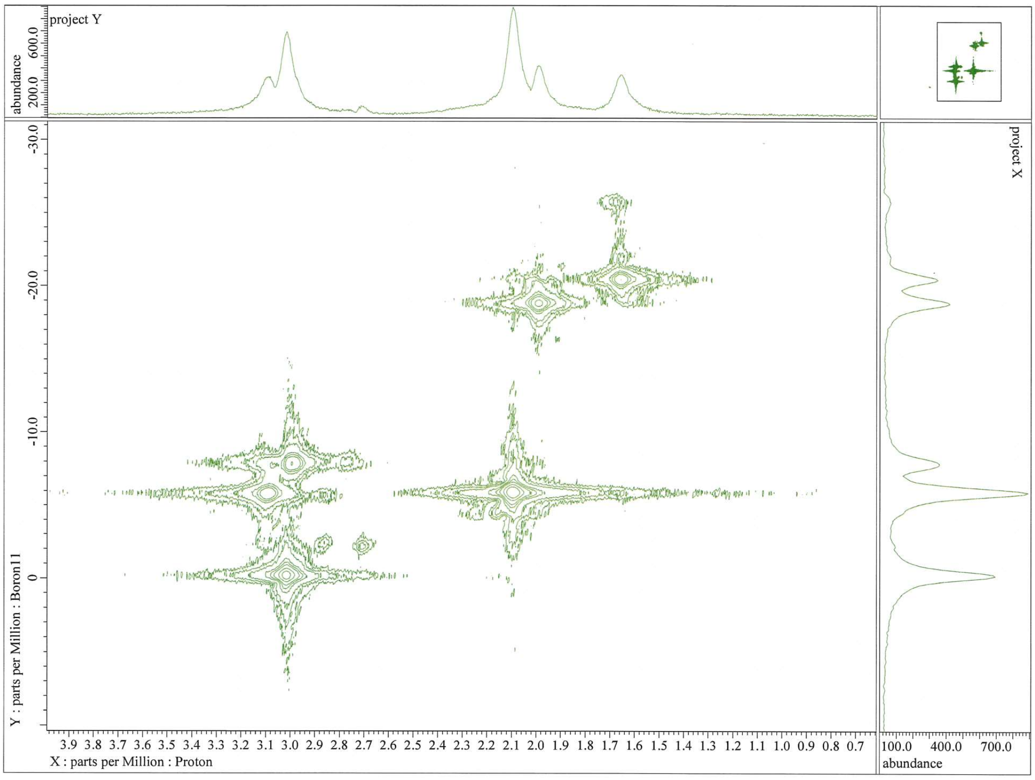

Figure S30. ^{1}H-^{11}B HMQC NMR spectrum of Cs[Br$_4$-**1**$^-$] – mixture of Cs[8,8´,9,9´-Br$_4$-**1**$^-$] and Cs[8,8´,12,12´-Br$_4$-**1**$^-$]. Based on this 2D experiment were assigned specific cluster hydrogens attached to borons.

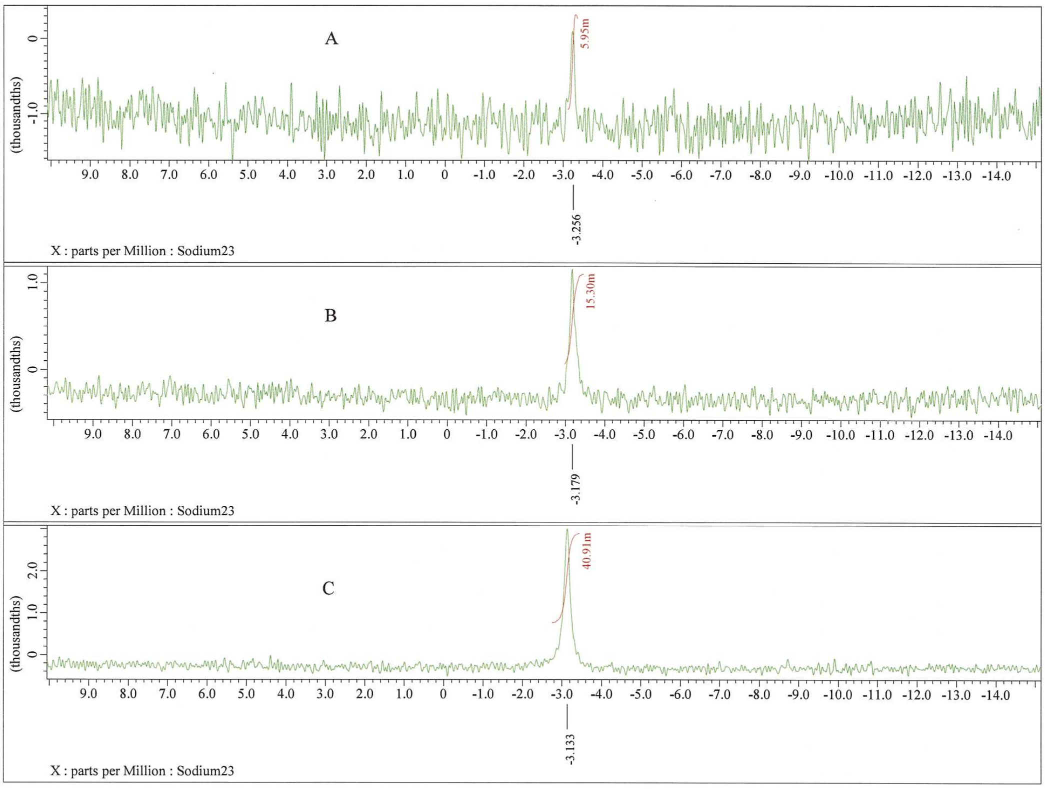

Figure S31. ^{23}Na NMR chemical shift peak area integrals increase as a proof of Na^+ extracted from the 3Å zeolite by hot methanol under argon atmosphere after 0 (A), 1 (B) and 4 (C) hours.

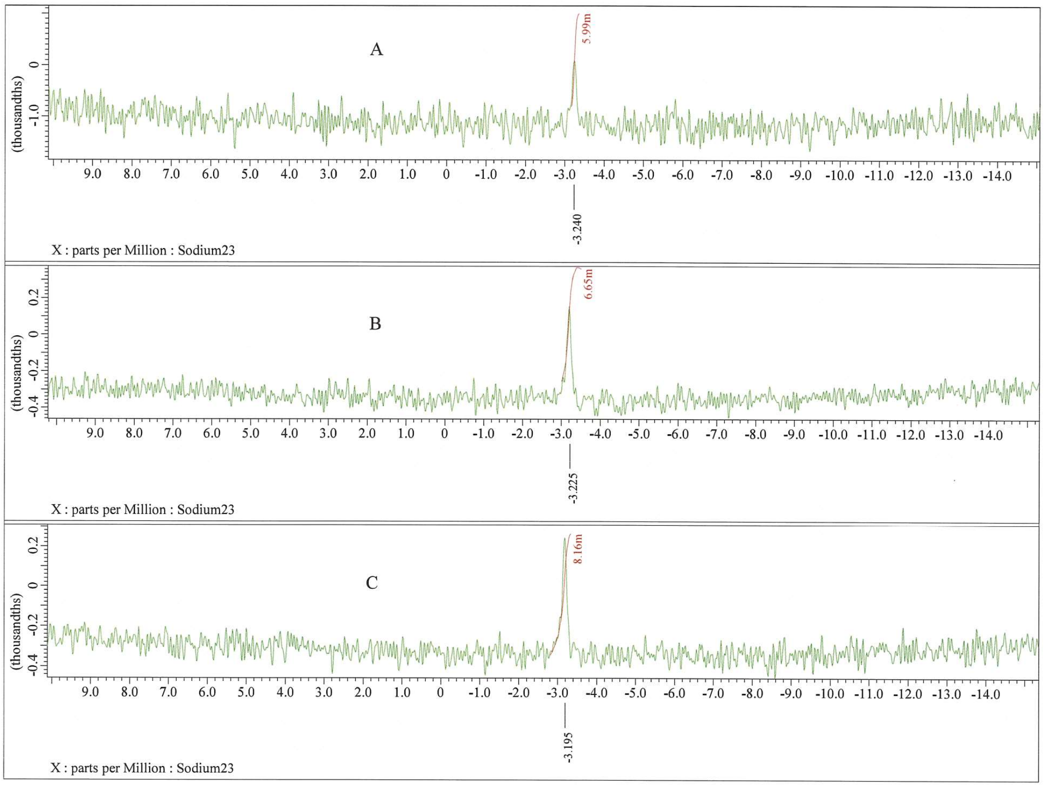

Figure S32. Comparative ^{23}Na NMR experiment of methanol refluxed under argon inert without zeolite in borosilicate glass apparatus after 0 (A), 1 (B) and 4 (C) hours.

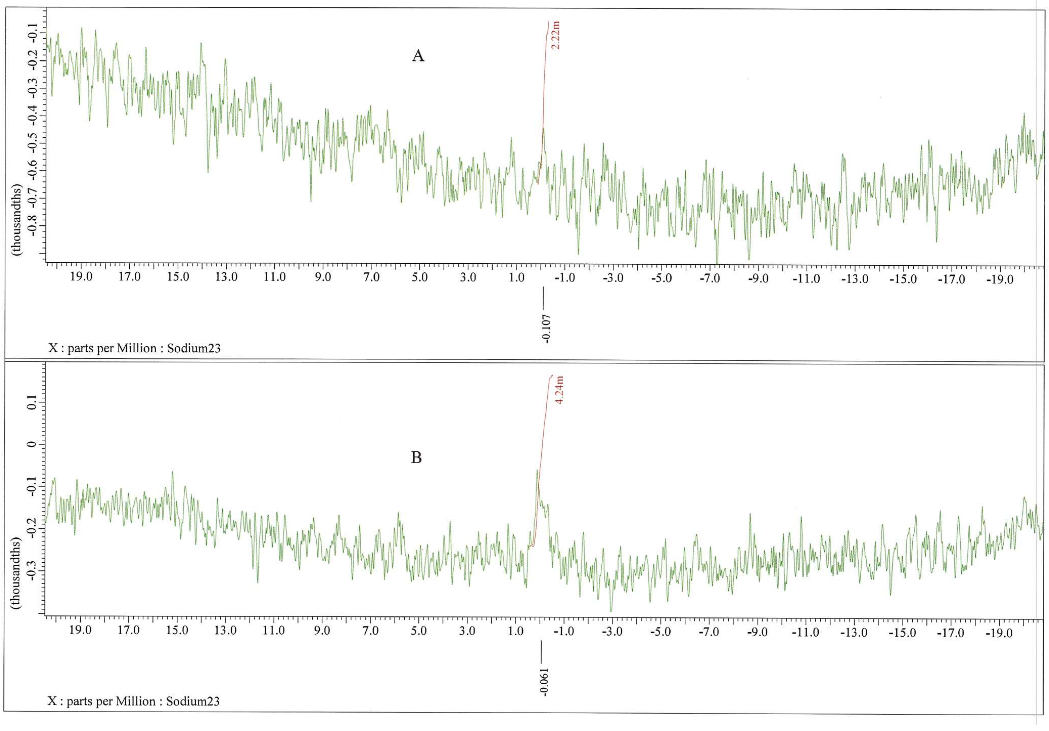

Figure S33. ^{23}Na NMR chemical shift peak area integrals increase as a proof of Na^+ extracted from the 3Å zeolite by hot ethanol under argon atmosphere after 0 (A) and 4 (B) hours.

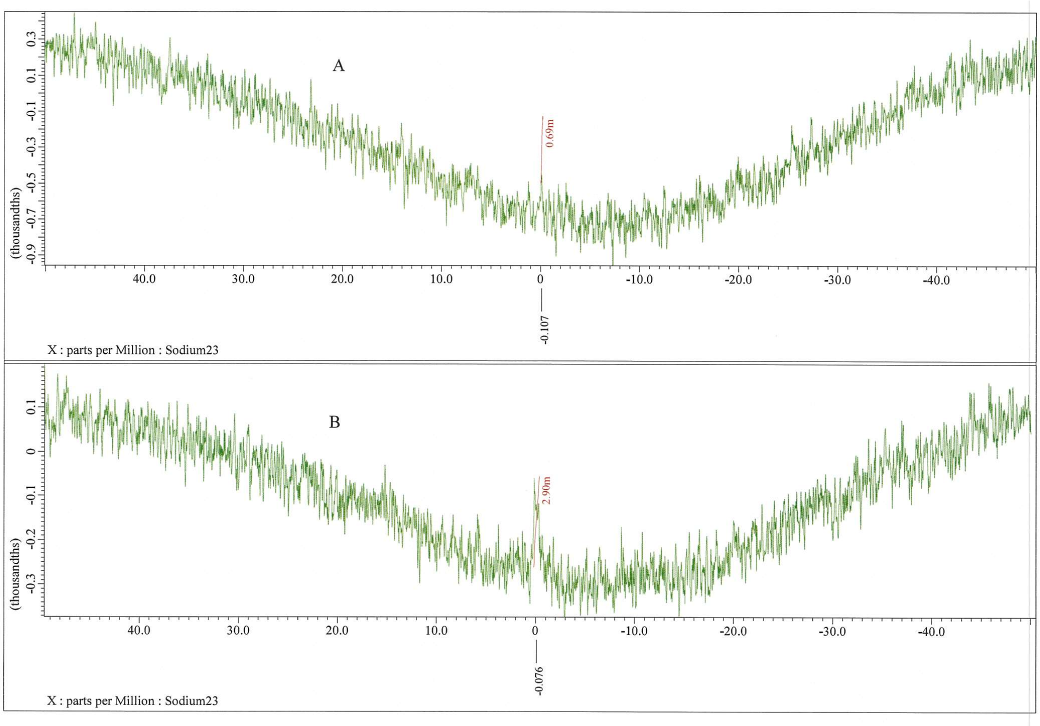

Figure S34. Comparative ^{23}Na NMR experiment of ethanol refluxed under argon inert in borosilicate glass apparatus without zeolite after 0 (A), 1 (B) and 4 (C) hours.
